# Insulin-like growth factor-1 attenuates oxidative stress-induced hepatocyte premature senescence in liver fibrogenesis via regulating nuclear p53–progerin interaction

**DOI:** 10.1038/s41419-019-1670-6

**Published:** 2019-06-06

**Authors:** Xiaoying Luo, Xiaoke Jiang, Jun Li, Yangqiu Bai, Zhen Li, Peiru Wei, Suofeng Sun, Yuan Liang, Shuangyin Han, Xiuling Li, Bingyong Zhang

**Affiliations:** 1 Department of Gastroenterology, Henan Provincial People’s Hospital, People’s Hospital of Zhengzhou University, Zhengzhou University, Zhengzhou, China; 2 Microbiome Laboratory, Henan Provincial People’s Hospital, People’s Hospital of Zhengzhou University, Zhengzhou University, Zhengzhou, China; 3 Department of Pathology, Henan Provincial People’s Hospital, People’s Hospital of Zhengzhou University, Zhengzhou University, Zhengzhou, China

**Keywords:** Mechanisms of disease, Liver fibrosis

## Abstract

Stress-induced premature senescence (SIPS), a state of cell growth arrest due to various stimuli, is implicated in the pathogeneses of hepatic fibrogenesis. Progerin, a permanently farnesylated mutant lamin A protein, likely leads to premature senescence to influent liver diseases. The previous reports showed that activation of insulin-like growth factor-1 (IGF-1) signaling could enhance cell longevity and attenuate liver fibrosis. However, the underlying mechanisms about hepatocyte premature senility in liver fibrosis, and how IGF-1 regulates cell premature aging and fibrogenesis, remain poorly understood. In the present study, we found the augment of hepatocyte oxidation and premature aging, along with the decrease of plasm IGF-1 level in patients with liver fibrosis and CCl_4_-induced liver injury rat models. Nevertheless, IGF-1 gene transfer to CCl_4_ rats to overexpress intrahepatic IGF-1 relieved hepatocyte oxidative stress and premature senescence, which was likely mediated by the p53/progerin pathway, to improve hepatic steatosis and fibrogenesis. In vitro, H_2_O_2_ caused abnormal accumulation of progerin in nuclear and activation of nuclear p53–progerin interaction to trigger primary rat hepatocyte premature senescence through the p21-independent pathway; while these effects were rescued by prolonged exogenous IGF-1 or the IGF-1 adenovirus vector. Furthermore, the IGF-1 adenovirus vector, transfected to H_2_O_2_-treated hepatocytes, reversed oxidative stress-induced premature senescence via enhancing cytoplasmic AKT1–p53 interaction and subsequently inhibiting nuclear p53–progerin interaction. Consequently, our data illuminate a novel role of IGF-1 in regulating stress-induced hepatocyte premature senescence in liver fibrosis: prolonged IGF-1 relieves oxidative stress-initiated hepatocyte premature senescence via inhibition of nuclear p53–progerin interaction to ameliorate hepatic steatosis and fibrogenesis.

## Introduction

Stress-induced premature senescence (SIPS), a kind of replicative senescence due to depletion of cell proliferative potential, is caused by a variety of sublethal stresses, such as H_2_O_2_, hypoxia, or hyperoxia^1^. Excessive stress-induced premature aging triggers abnormal accumulation of senescent like-cells to affect cell function, tissue repair, as well as aging-related disorders and diseases. Recent studies have provided critical insights on the close relation between premature senescence and liver diseases. The senescence of intrahepatic cells, including hepatocytes, hepatic stellate cells (HSCs), and hepatic sinus endothelial cells (HSECs), is observed in liver fibrosis^2^. Besides, some disease paradigms confirmed that cirrhosis in Werner’s syndrome was a manifestation of premature senescence^3^. Moreover, during aging, oxidative stress-induced mitochondrial DNA mutations triggered a vicious cycle with increasing production of reactive oxygen species (ROS) to accelerate liver ballooning degeneration^4^. These results imply that oxidative stress causes cell premature aging to aggravate liver injury. Hence, elucidation of the underlying mechanisms for cell premature senility may be a key to our understanding of liver fibrosis pathogenesis.

Progerin, a mutant prelamin A protein, leads to cell proliferative arrest and premature aging to promote cell dysfunction. The abnormal accumulation of progerin initiates alteration of nuclear morphology (manifested as an accelerated aging phenotype)^5^. Normally, the production of mature lamin A is a finely cell-based system; whereas perturbing maturation of prelamin A facilitates cells into an early onset of aging phenotype^6,7^. Nonetheless, whether elevated expression and abnormally distribution of progerin are responsible for hepatocyte premature senescence in liver fibrosis; and more importantly, these mechanisms remain unclear.

Insulin-like growth factor 1 (IGF-1), known as a cytoprotective hormone, mainly synthesized in liver, regulates cell senescence, survival, proliferation, and so on^8–10^. In cirrhosis, reduction or deficiency of IGF-1 plays a key role in intrahepatic metabolic disorders^11^. It is intriguing that both recombinant IGF-1 (rIGF-1) and viral vectors encoding IGF-1 treatment could promote hepatoprotective activities in liver injury animal models and cirrhotic patients^11–15^. These findings indicate that IGF-1 therapy might be considered for amelioration of cell premature aging and liver fibrosis.

Hence, progerin, a pivotal protein, was likely linked to oxidative stress-induced hepatocyte premature senility for facilitating liver fibrosis; and IGF-1 may influent progerin-related pathway to attenuate premature senescence and fibrosis. Our present study aims to investigate the role of IGF-1 in hepatocyte premature senescence in liver fibrosis mediated by progerin.

## Results

### Elevated nuclear p53 and progerin are closely related to cell premature senescence in liver fibrosis patients

**Fig. 1:**
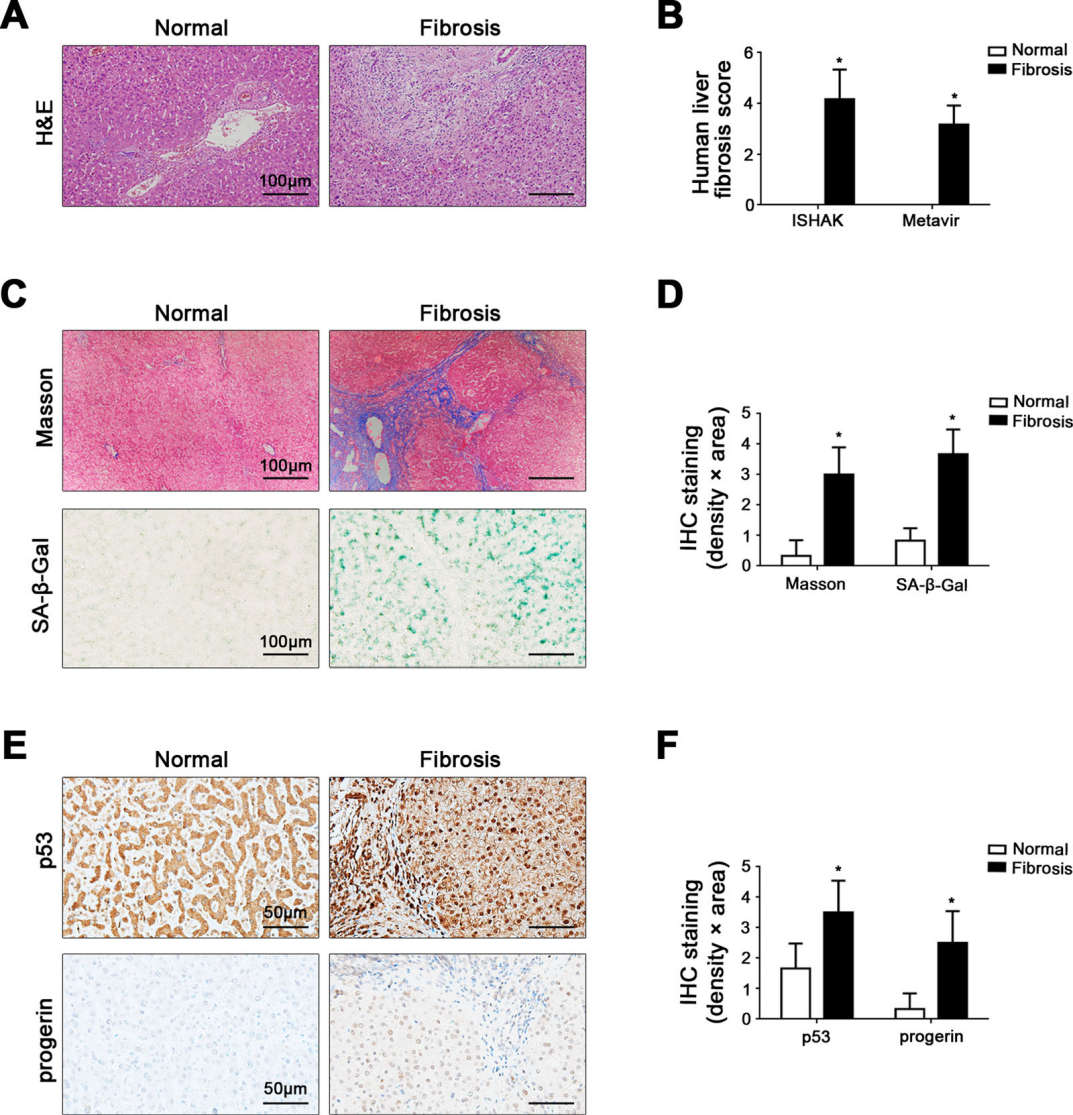
Elevated nuclear p53 and progerin are closely related to cell premature senescence in liver fibrosis patients. **a** The H&E in liver biopsy specimens of patients (Scale bar: 100 μm). **b** The quantified analysis of liver fibrosis with ISHAK and Metavir score. *P < 0.05 versus the normal group. **c** Masson staining and SA-β-gal staining in liver biopsy specimens (Scale bar: 100 μm). SA-β-gal positive cells, which implied senescent cells, were blue stained. **d** The semiquantified analysis of Masson staining and SA-β-gal staining. *P < 0.05 versus the normal group. **e** The immunohistochemical staining (IHC) for p53 and progerin in liver biopsy specimens (Scale bar: 50 μm). **f** The semiquantitative score of IHC staining for p53 and progerin. *P < 0.05 versus the normal group

Compared with the normal group, the fibrosis score, as well as the area density of Masson staining and senescence-associated β-galactosidase (SA-β-gal) staining in human liver fibrotic tissue were dramatically higher (Fig. 1a–d). Intriguingly, p53 and progerin in cell nuclei highly expressed in human liver fibrotic tissue (Fig. 1e, f). These suggested that the increased expression of nuclear p53 and progerin in intrahepatic cells could be strongly linked to cell premature senescence in human liver fibrosis.

### Hepatocyte premature senescence is initiated by oxidative stress, along with reduction of IGF-1 during CCl_4_-induced liver fibrogenesis

The area density of Masson staining and SA-β-gal staining showed that with time, CCl_4_ initiated hepatic steatosis on Day 6 and the early stage of fibrosis on Day 28; meanwhile, the senescence of intrahepatic cells was augmented significantly (Supplementary Fig. 1a–c). In addition, there was a time-dependent increase of H_2_O_2_ content, along with the high expression of p53 and p21 in primary hepatocytes of CCl_4_-induced rats (Supplementary Fig. 1d, e). These data suggested that oxidative stress activated the p53-dependent hepatocyte senescence during the first stage of CCl_4_-induced liver fibrosis. Besides, the protein levels of progerin and Lamin A/C in primary rat hepatocytes were constantly upregulated, confirming hepatocyte premature senility in CCl_4_-induced acute and chronic liver injury (Supplementary Fig. 1d). However, there was a time-dependent downregulation of plasm IGF-1 content in the rat models (Supplementary Fig. 1f). Therefore, these results suggested that during liver steatosis and fibrogenesis in vivo, CCl_4_-induced oxidative stress, with the decrease of IGF-1, likely initiated hepatocyte premature senescence mediated by p53.

### IGF-1 gene transfer to CCl_4_-induced rat models alleviates hepatocyte premature senescence and fibrogenesis

**Fig. 2:**
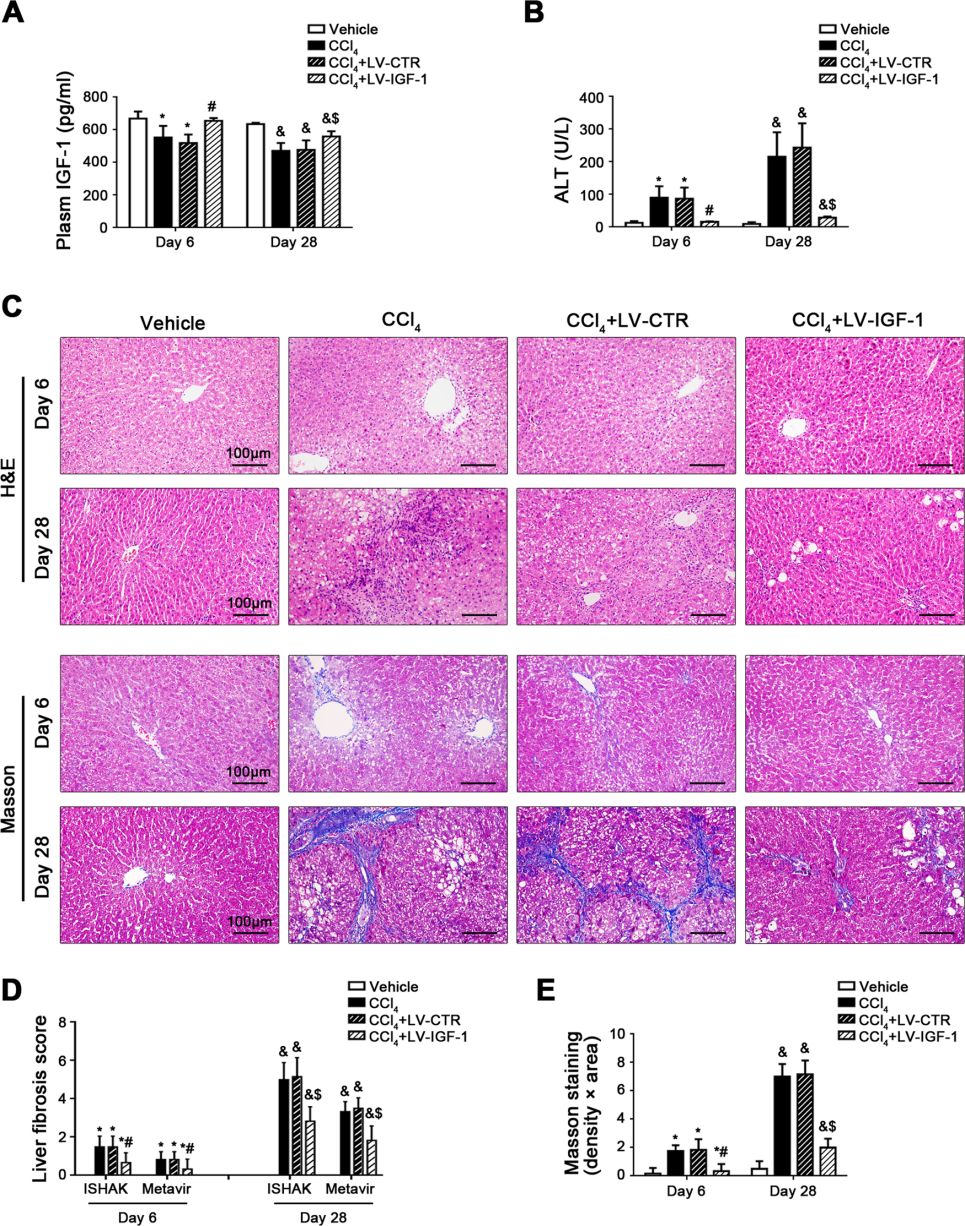
IGF-1 gene transfer to CCl_4_-induced rat models alleviates hepatic steatosis and fibrogenesis. **a** The plasm IGF-1 content of CCl_4_-induced rat models on Day 6 and Day 28. *P < 0.05 versus the Vehicle group on Day 6; ^#^*P* < 0.05 versus the CCl_4_ + LV-CTR group on Day 6; ^&^*P* < 0.05 versus the Vehicle group on Day 28; ^$^*P* < 0.05 versus the CCl_4_ + LV-CTR group on Day 28. **b** The ALT content of CCl_4_-induced rat models on Day 6 and Day 28. *P < 0.05 versus the Vehicle group on Day 6; ^#^*P* < 0.05 versus the CCl_4_ + LV-CTR group on Day 6; ^&^*P* < 0.05 versus the Vehicle group on Day 28; ^$^*P* < 0.05 versus the CCl_4_ + LV-CTR group on Day 28. **c** The H&E and Masson staining in liver biopsy specimens of CCl_4_-induced rat models on Day 6 and Day 28. (Scale bar: 100 μm). **d** The quantitation with ISHAK and Metavir score in liver biopsy specimens of CCl_4_-induced rat models on Day 6 and Day 28. *P < 0.05 versus the Vehicle group on Day 6; ^#^*P* < 0.05 versus the CCl_4_ + LV-CTR group on Day 6; ^&^*P* < 0.05 versus the Vehicle group on Day 28; ^$^*P* < 0.05 versus the CCl_4_ + LV-CTR group on Day 28. **e** The area density of Masson staining in rat liver tissue on Day 6 and Day 28. *P < 0.05 versus the Vehicle group on Day 6; ^#^*P* < 0.05 versus the CCl_4_ + LV-CTR group on Day 6; ^&^*P* < 0.05 versus the Vehicle group on Day 28; ^$^*P* < 0.05 versus the CCl_4_ + LV-CTR group on Day 28. *n* = 6 per group

**Fig. 3:**
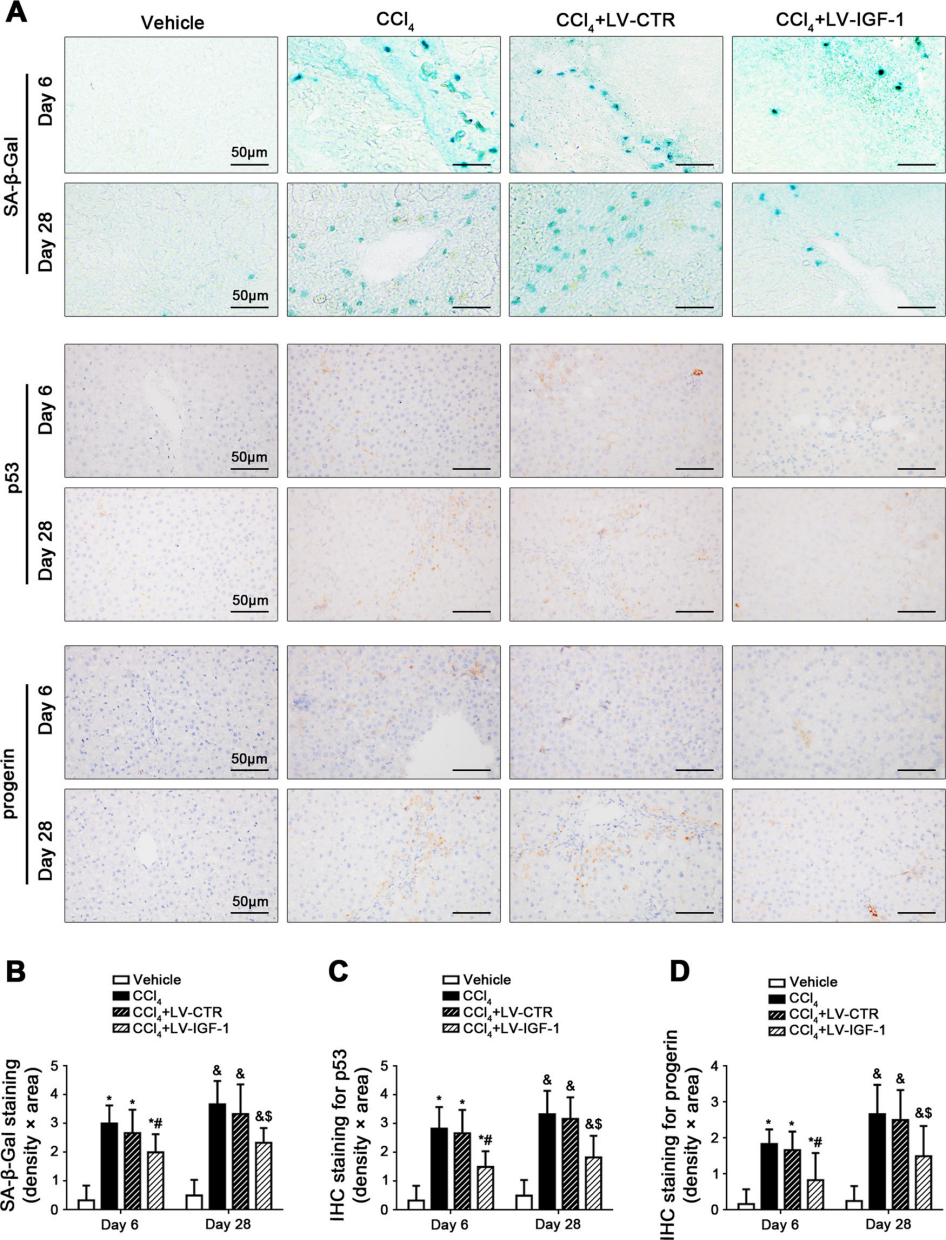
IGF-1 gene transfer to CCl_4_-induced rat models alleviates hepatocyte premature senescence. **a** The SA-β-gal staining, as well as the IHC staining for p53 and progerin in liver biopsy specimens of CCl_4_-induced rat models on Day 6 and Day 28. (Scale bar: 50 μm). **b** The semiquantitative score of SA-β-gal staining of rat liver tissue on Day 6 and Day 28. *P < 0.05 versus the Vehicle group on Day 6; ^#^*P* < 0.05 versus the CCl_4_ + LV-CTR group on Day 6; ^&^*P* < 0.05 versus the Vehicle group on Day 28; ^$^*P* < 0.05 versus the CCl_4_ + LV-CTR group on Day 28. **c** The semiquantitative score of IHC staining for p53 of rat liver tissue on Day 6 and Day 28. *P < 0.05 versus the Vehicle group on Day 6; ^#^*P* < 0.05 versus the CCl_4_ + LV-CTR group on Day 6; ^&^*P* < 0.05 versus the Vehicle group on Day 28; ^$^*P* < 0.05 versus the CCl_4_ + LV-CTR group on Day 28. **d** The semiquantitative score of IHC staining for progerin of rat liver tissue on Day 6 and Day 28. *P < 0.05 versus the Vehicle group on Day 6; ^#^*P* < 0.05 versus the CCl_4_ + LV-CTR group on Day 6; ^&^*P* < 0.05 versus the Vehicle group on Day 28; ^$^*P* < 0.05 versus the CCl_4_ + LV-CTR group on Day 28. *n* = 6 per group

To evaluate the effect of IGF-1 on hepatocyte premature senescence and liver fibrosis, IGF-1 lentiviral vector was transferred to CCl_4_-induced rat models to overexpress IGF-1 of liver. As expected, plasm IGF-1 level on Day 6 and Day 28 was remarkably increased in the CCl_4_ + LV-IGF-1 group, as compared to the CCl_4_ group and the CCl_4_ + LV-CTR group (Fig. 2a). Meanwhile, compared with the CCl_4_ + LV-CTR group, the IGF-1R expression was elevated in hepatocytes, HSCs, and HSECs of the CCl_4_ + LV-IGF-1 group (Supplementary Fig. 2a). There were about 80% of hepatocytes infected after injection (Supplementary Fig. 2b, c). Moreover, overexpression of IGF-1 with lentiviral vector attenuated the content of serum glutamic pyruvic transaminase (ALT) on Day 6 and Day 28 and relieved CCl_4_-induced hepatic steatosis and fibrogenesis (Fig. 2b–e), confirming alleviation of CCl_4_-induced acute and chronic liver injury by overexpressing IGF-1. In addition, SA-β-gal highly expressed, together with elevated expression of p53 and progerin in cell nuclei in the CCl_4_ group and the CCl_4_ + LV-CTR group on Day 6 and Day 28; however, these effects were rescued by IGF-1 lentiviral vector (Fig. 3a–d), implying that CCl_4_-induced p53-dependent cell premature senescence were improved by IGF-1 gene therapy.

**Fig. 4:**
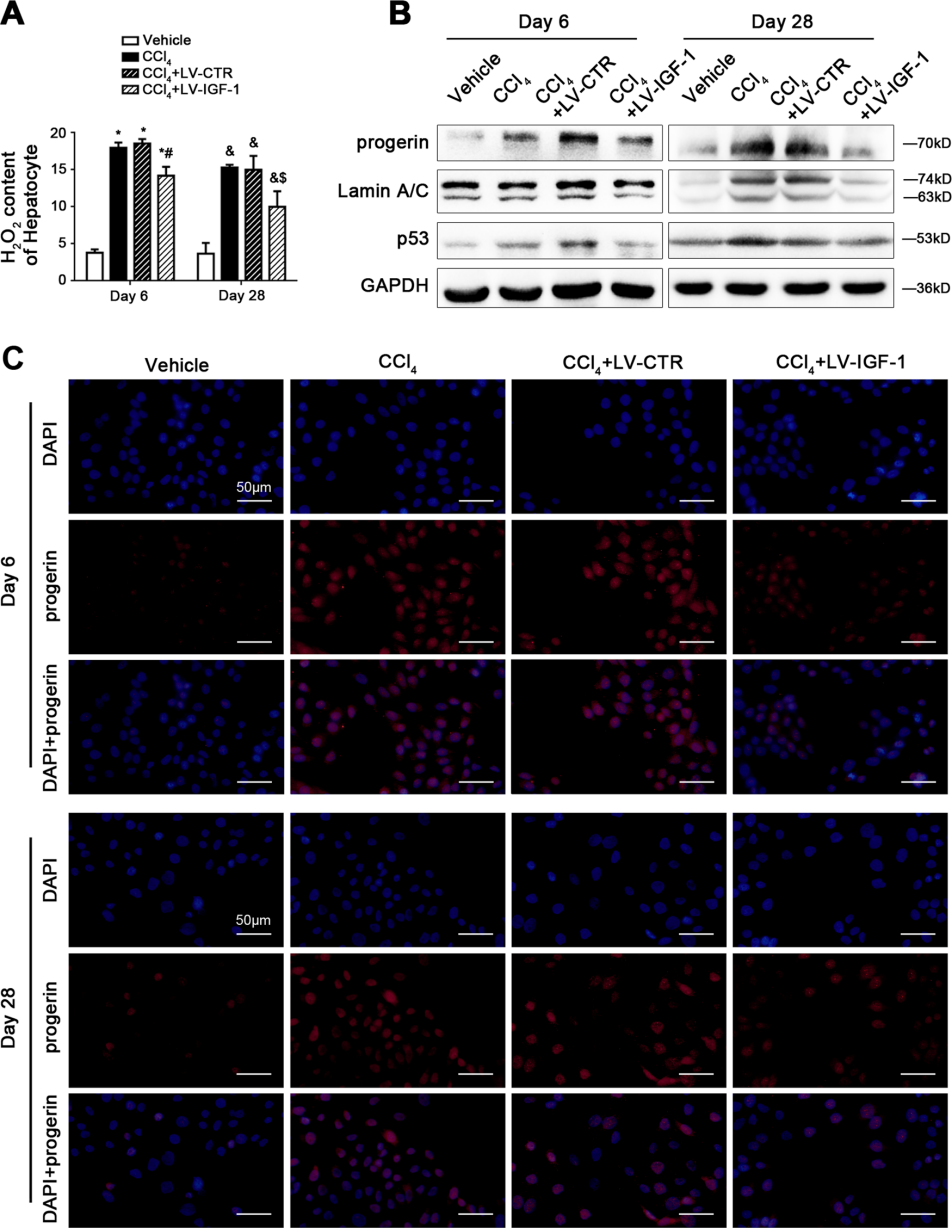
IGF-1 gene transfer to CCl_4_-induced rat models reduces nuclear progerin and p53 protein levels of primary rat hepatocytes. **a** The H_2_O_2_ content of primary rat hepatocytes, isolated from CCl_4_-induced rat models on Day 6 and Day 28. *P < 0.05 versus the Vehicle group on Day 6; ^#^*P* < 0.05 versus the CCl_4_ + LV-CTR group on Day 6; ^&^*P* < 0.05 versus the Vehicle group on Day 28; ^$^*P* < 0.05 versus the CCl_4_ + LV-CTR group on Day 28. **b** Representative immunoblots of progerin, Lamin A/C, and p53 of primary hepatocytes isolated from the CCl_4_-induced rat models on Day 6 and Day 28. **c** The progerin protein expression (red) in hepatocytes of the four groups (Vehicle, CCl_4_, CCl_4_ + LV-CTR, CCl_4_ + LV-IGF-1) on Day 6 and Day 28, shown by immunofluorescence. Nuclear were showed by DAPI (blue). Scale bar: 50 μm. *n* = 6 per group

In order to characterize hepatocytes responding to IGF-1 overexpression in CCl_4_-induced hepatic steatosis and fibrosis, we detected oxidation and aging-related markers in purified primary hepatocytes, which isolated from rat model livers. We found that the H_2_O_2_ content of hepatocytes was decreased in IGF-1 lentiviral vector-treated models, compared to the CCl_4_ group and the CCl_4_ + LV-CTR group (Fig. 4a), indicating that CCl_4_-induced hepatocyte oxidative stress was inhibited by IGF-1 overexpression. Furthermore, the protein levels of progerin, Lamin A/C, and p53 in primary hepatocytes on Day 6 and Day 28 were also predominantly downregulated by IGF-1 overexpression (Fig. 4b). In agreement with these data, the immunofluorescence showed that on Day 6 and Day 28, compared with the vehicle group, progerin highly expressed in the nuclear area of hepatocytes in the CCl_4_ group and the CCl_4_ + LV-CTR group; in contrast, less progerin expressed in hepatocytes of the CCl_4_ + LV-IGF-1 group (Fig. 4c), suggested that hepatocyte premature aging was likely triggered due to CCl_4_-induced oxidative stress mediated by p53, which was rescued by IGF-1 gene therapy.

Taken together, these data indicated that IGF-1 lentiviral vector could transduce the rat liver and overexpress functional IGF-1. IGF-1 gene therapy might alleviate hepatocyte premature senescence through regulating the p53/progerin pathway, to improve hepatic steatosis and fibrogenesis.

### H_2_O_2_ induces hepatocyte premature senescence via the p53/progerin-dependent pathway

To further delineate the molecular mechanism of oxidative stress-induced cell premature senescence, primary hepatocytes, isolated from normal rats, were cultured and stimulated with H_2_O_2_ at the different doses (0, 10, 50, 100, 200 nM) for 24 h or at the dose (200 nM) from 0 to 24 h. On the other hand, hepatocytes were transfected with p53 siRNA, progerin siRNA, p21 siRNA, or nontarget siRNA (called NC), and then administered with H_2_O_2_ (200 nM) for 24 h. As expected, with time or with increase of H_2_O_2_ concentration, the protein levels of progerin, p53, and p21 in primary rat hepatocytes showed a concentration-dependent and a time-dependent upregulation of progerin expression and activation of the p53/p21 pathway, as a result of oxidative stress (Supplementary Fig. 3a, b).

**Fig. 5:**
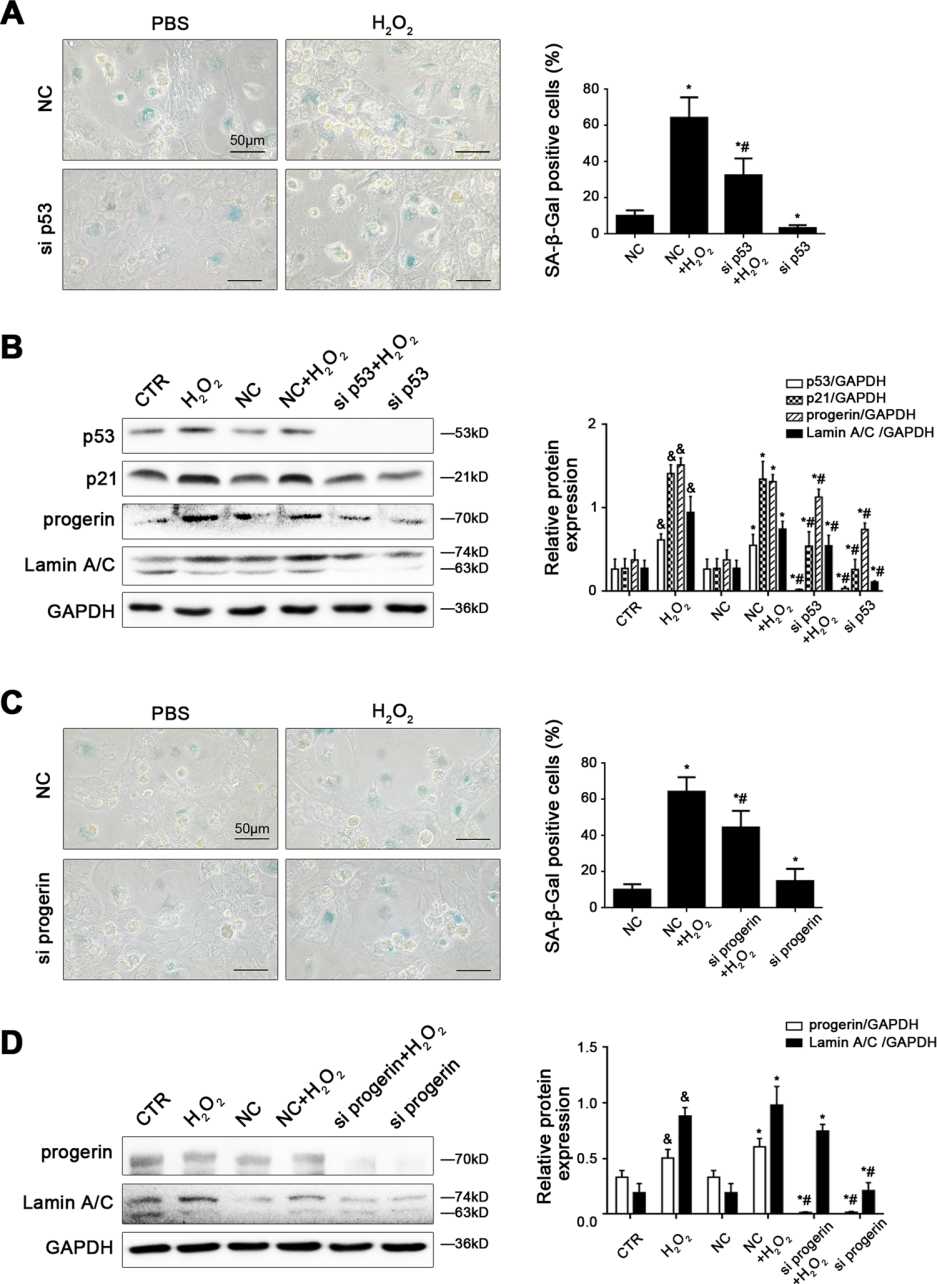
H_2_O_2_ induces hepatocyte premature senescence via the p53/progerin-dependent pathway. Freshly hepatocytes, isolated from normal rats and cultured in vitro, were transfected with p53 siRNA, progerin siRNA, or nontarget siRNA (called NC), and then administered with H_2_O_2_ (200 nM) for 24 h. **a** SA-β-gal activity in primary rat hepatocytes of the four groups (NC, NC + CCl_4_, CCl_4_ + si p53, si p53), was revealed by SA-β-gal staining. Scale bar: 50 μm. SA-β-gal positive cells are quantified in the graph, right. *P < 0.05 versus the NC group; ^#^*P* < 0.05 versus the NC + H_2_O_2_ group. **b** Representative immunoblots of p53, p21, progerin, and Lamin A/C of primary rat hepatocytes of the six groups (CTR, H_2_O_2_, NC, NC + CCl_4_, CCl_4_ + si p53, and si p53). The relative protein expression is quantified in the graph, right. ^&^*P* < 0.05 versus the CTR group; ^*^P < 0.05 versus the NC group; ^#^*P* < 0.05 versus the NC + H_2_O_2_ group. **c** SA-β-gal activity in primary rat hepatocytes of the four groups (NC, NC + CCl_4_, CCl_4_ + si progerin, and si progerin), was revealed by SA-β-gal staining. Scale bar: 50 μm. SA-β-gal positive cells are quantified in the graph, right. *P < 0.05 versus the NC group; ^#^P < 0.05 versus the NC + H_2_O_2_ group. **d** Representative immunoblots of progerin and Lamin A/C of primary rat hepatocytes of the six groups (CTR, H_2_O_2_, NC, NC + CCl_4_, CCl_4_ + si progerin, and si progerin). The relative protein expression is quantified in the graph, right. ^&^*P* < 0.05 versus the CTR group; *P < 0.05 versus the NC group; ^#^*P* < 0.05 versus the NC + H_2_O_2_ group

However, the evident activity of SA-β-gal, caused by H_2_O_2_, was remarkably reduced by silencing p53 with p53 siRNA, silencing progerin with progerin siRNA, or silencing p21 with p21 siRNA (Fig. 5a, c; Supplementary Fig. 4a). In addition, knockdown of p53 with siRNA diminished H_2_O_2_-induced high expression of p53, progerin, Lamin A/C and p21; meanwhile, knockdown of progerin with siRNA decreased H_2_O_2_-induced elevated expression of Lamin A/C, except p21 (Fig. 5b, d). Nevertheless, silencing p21 with siRNA did not alter the H_2_O_2_-induced high expression of progerin and Lamin A/C (Supplementary Fig. 4b). These results indicated that H_2_O_2_ induced hepatocyte senescence mediated by p53 and p21; but oxidative stress-induced premature aging through the p53/progerin-dependent pathway, which is the p21-independent pathway.

### IGF-1 therapy relieves hepatocyte premature senescence via inhibiting nuclear p53–progerin interaction

To further reveal the role of IGF-1 in oxidative stress-induced premature aging of hepatocytes, firstly, we explored whether short-term or prolonged exogenous IGF-1 treatment attenuated hepatocyte premature aging. Primary hepatocytes, isolated from normal rat livers, treated with H_2_O_2_ (200 nM) for 24 h, were simultaneously administered with IGF-1 (100 ng/ml) for 1 and 24 h. Compared with the H_2_O_2_ group, short-term exogenous IGF-1 treatment for 1 h did not alter the protein levels of p53 and progerin, as well as the activity of SA-β-gal, though p21 protein expression was downregulated. These data implied that short-term IGF-1 treatment did not alter oxidative stress-induced hepatocyte premature senescence. However, prolonged exogenous IGF-1 suppressed the p53/progerin pathway to diminish hepatocyte premature senescence through activating the PI3K/AKT1 pathway (Supplementary Fig. 5a, b).

**Fig. 6:**
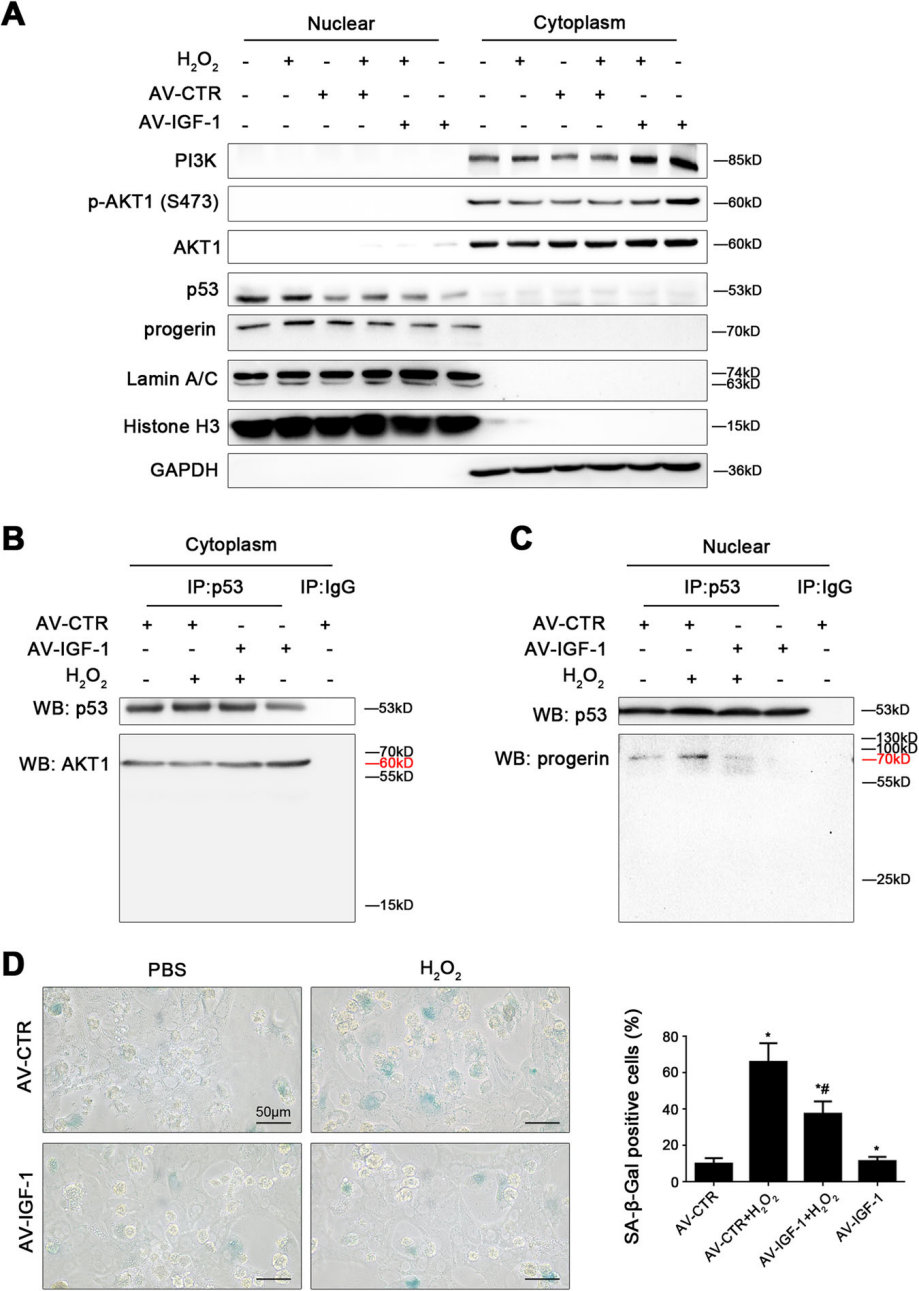
Overexpressing IGF-1 with adenovirus vector relieves hepatocyte premature aging through activating the PI3K-AKT1 pathway and disrupting nuclear p53–progerin interaction. Freshly hepatocytes, isolated from normal rats and cultured in vitro, were transfected the IGF-1 adenovirus vector to overexpress IGF-1 (called AV-IGF-1) or nontarget adenovirus vector (called AV-CTR), and then treated with H_2_O_2_ (200 nM) for 24 h. And then we extracted nuclear and cytoplasmic protein of rat hepatocytes, and detected their protein levels. **a** Representative immunoblots of PI3K, p-AKT1, AKT1, p53, progerin, and Lamin A/C in nuclei and cytoplasm of primary rat hepatocytes of six groups (CTR, H_2_O_2_, AV-CTR, AV-CTR + H_2_O_2_, AV-IGF-1 + H_2_O_2_, and AV-IGF-1). **b** Interaction of cytoplasmic p53 with AKT1 was detected by the co-IP assay. Cytoplasmic p53 of primary rat hepatocytes were individually immunoprecipitated, and p53 and AKT1 subjected to immunoblotting analysis as indicated. **c** Interaction of nuclear p53 with progerin was detected by the co-IP assay. Nuclear p53 of primary rat hepatocytes were individually immunoprecipitated, as well as p53 and progerin subjected to immunoblotting analysis as indicated. **d** SA-β-gal activity in rat hepatocytes of the four groups (AV-CTR, AV-CTR + H_2_O_2_, AV-IGF-1 + H_2_O_2_, and AV-IGF-1), was revealed by SA-β-gal staining. Scale bar: 50 μm. SA-β-gal positive cells are quantified in the graph, right. *P < 0.05 versus the AV-CTR group; ^#^*P* < 0.05 versus the AV-CTR + H_2_O_2_ group

**Fig. 7:**
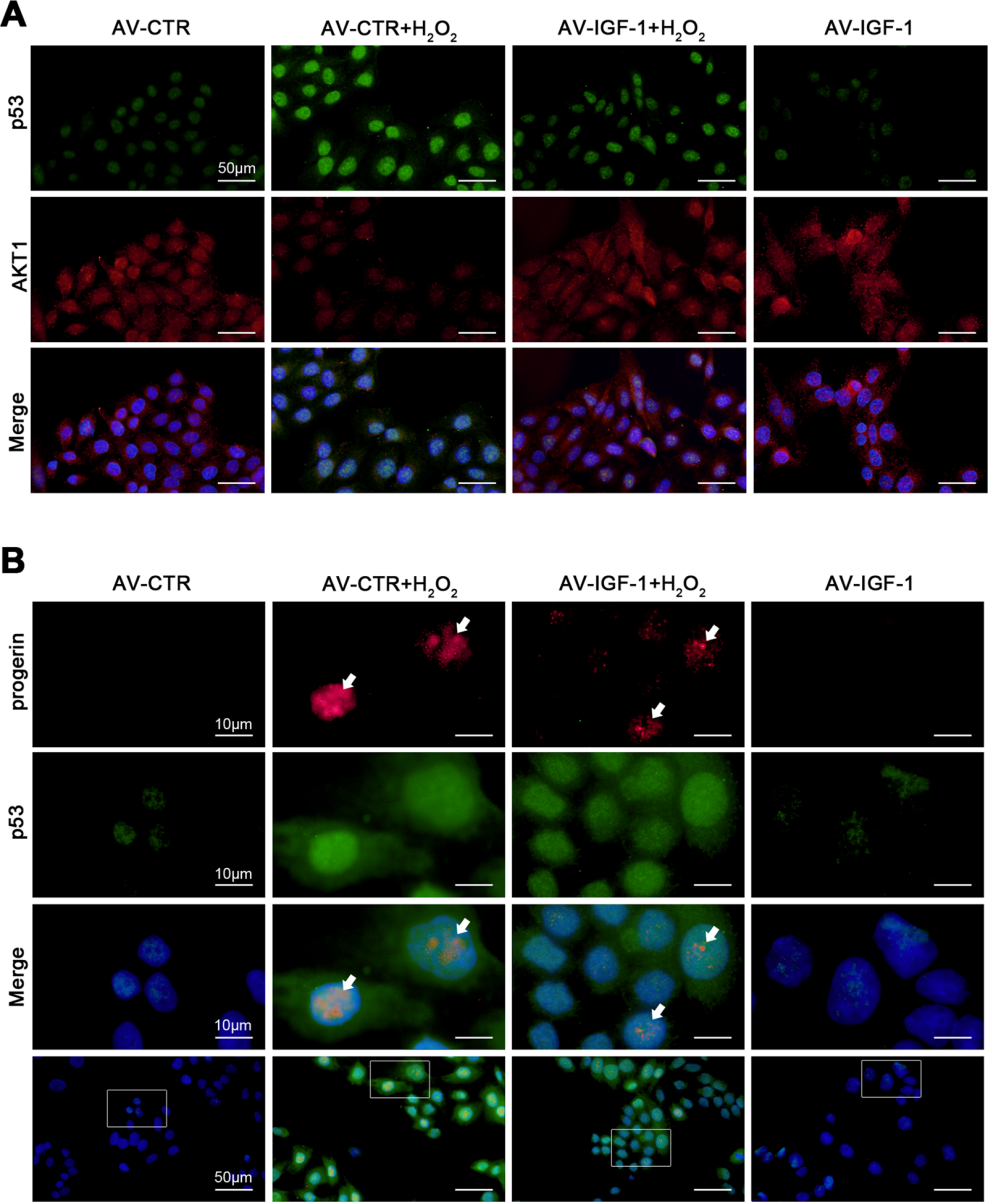
Overexpressing IGF-1 with adenovirus vector inhibits p53 nuclear translocation via strengthening AKT1–p53 interaction. Freshly hepatocytes, isolated from normal rats and cultured in vitro, were transfected the IGF-1 adenovirus vector to overexpress IGF-1 (called AV-IGF-1) or nontarget adenovirus vector (called AV-CTR), and then treated with H_2_O_2_ (200 nM) for 24 h. **a** The co-localization of p53 (green) with AKT1 (red) in hepatocytes of four groups (AV-CTR, AV-CTR + H_2_O_2_, AV-IGF-1 + H_2_O_2_, and AV-IGF-1), shown by immunofluorescence. Scale bar: 50 μm. **b** The co-localization of p53 (green) with progerin (red) in hepatocytes of four groups (AV-CTR, AV-CTR + H_2_O_2_, AV-IGF-1 + H_2_O_2_, and AV-IGF-1), shown by immunofluorescence (Scale bar: 10 and 50 μm)

Then, to ulteriorly determine how IGF-1 gene overexpression alleviated SIPS of hepatocytes, primary hepatocytes were transfected the IGF-1 adenovirus vector to overexpress IGF-1 (namely AV-IGF-1) or nontarget adenovirus vector (called AV-CTR), and then treated with H_2_O_2_ (200 nM) for 24 h. We found that the protein expression of PI3K, p-AKT1 (S473), AKT1, and p53 in nuclei and cytoplasm showed that H_2_O_2_ increased nuclear p53 protein level but decreased the protein levels of PI3K, p-AKT1 (S473), and AKT1; in contrast, this effect was rescued by overexpression of IGF-1 with adenovirus vector via activating the PI3K/AKT1 pathway in cytoplasm (Fig. 6a). In addition, the co-immunoprecipitation (Co-IP) assay revealed that IGF-1 adenovirus vector enhanced the co-precipitation of AKT1 with p53 in cytoplasm of hepatocytes (Fig. 6b); meanwhile, the immunofluorescence for the localization of p53 and AKT1, showed more accumulation of p53 protein in nuclear area and less AKT1 protein expression in the AV-CTR + H_2_O_2_ group; while AKT1 highly expressed in the AV-IGF-1 group and the AV-IGF-1 + H_2_O_2_ group (Fig. 7a). These data demonstrated that more p53 likely translocated from cytoplasm to nucleus due to H_2_O_2_-induced oxidative stress; whereas overexpression of IGF-1 with adenovirus vector inhibited the p53 nuclear translocation via activating the PI3K/AKT1 pathway and subsequently enhanced the cytoplasm AKT1–p53 interaction.

However, the protein expression of progerin and Lamin A/C in nuclei and cytoplasm showed that progerin and Lamin A/C highly expressed in nuclei of hepatocytes in the AV-CTR + H_2_O_2_ group, which were diminished by overexpression of IGF-1 with adenovirus vector (Fig. 6a). Furthermore, the co-IP assay displayed that H_2_O_2_ increased the co-precipitation of p53 with progerin in nuclei of hepatocytes; whereas overexpression of IGF-1 with adenovirus vector disrupted this interaction (Fig. 6c). In addition, p53 co-localized with progerin in nuclear area of hepatocytes in the AV-CTR + H_2_O_2_ group; on the contrary, less co-localization of progerin with p53 were displayed in the AV-IGF-1 + H_2_O_2_ group (Fig. 7b). These results indicated that H_2_O_2_ initiated more p53 nuclear translocation and intensified the nuclear p53–progerin interaction, which broke by overexpression of IGF-1 with adenovirus vector. As expected, the IGF-1 adenovirus vector to overexpress IGF-1 also relieved H_2_O_2_-induced hepatocyte premature senescence (Fig. 6d).

Hence, prolonged IGF-1 or IGF-1 gene therapy could disrupt the nuclear p53–progerin interaction to repress oxidative SIPS via activating the PI3K/AKT1 pathway and inhibiting p53 nuclear translocation.

## Discussion

**Fig. 8:**
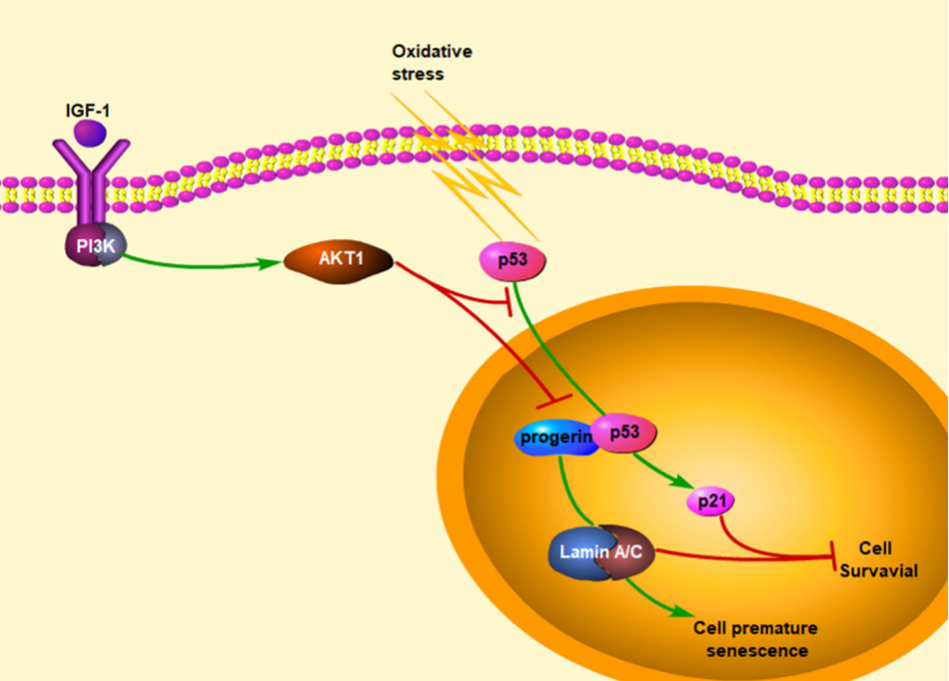
A schematic view of major signal transduction pathways, involves in the conclusion that prolonged IGF-1 diminishes hepatocyte premature senescence to relief liver fibrogenesis via disrupting nuclear p53–progerin interaction

In the present study, we demonstrated that permanent IGF-1 attenuated oxidative SIPS of hepatocytes via inhibiting the interaction between nuclear p53 with progerin (Fig. 8). The principal findings include the followings: (1) During CCl_4_-induced hepatic steatosis and fibrosis, hepatocyte premature senescence initiated by oxidative stress; whereas IGF-1 gene therapy suppressed these effects to improve hepatic steatosis and fibrogenesis. (2) H_2_O_2_ caused hepatocyte premature senility via activating the p53/progerin pathway, which was the p21-independent way. (3) Oxidative stress reinforced the nuclear p53–progerin interaction to accelerate hepatocyte premature senescence; in contrast, IGF-1 overexpression by prolonged exogenous IGF-1 or IGF-1 adenovirus vector, inhibited the p53 nuclear translocation via activation of the PI3K/AKT1 pathway, and subsequently reduced the nuclear p53–progerin interaction to relief premature senescence.

Cellular senescence, a state of permanent inhibition of cell proliferation, closely links to aging and aging-related diseases^16,17^. SIPS, a kind of specific senescence, is caused by stimuli including oxidative stress to halt cell growth. SIPS plays a key role in regulating cell metabolism and function^18^. Stressed cells in premature senescence share some common features with cells in natural aging: aging-like phenotype, the activation of senescence-associated β-galactosidase (SA-β-gal), cell cycle arrest, the disorder of gene expression, and telomere shortening^1^. Recently, it’s reported that intrahepatic cell senescence strongly associated with the pathogenesis of liver diseases, such as steatohepatitis, liver fibrosis, and cirrhosis. Werner’s syndrome is a type of condition, in which patients undergoing premature aging; and some disease paradigms confirm that liver cirrhosis in Werner’s syndrome is a manifestation of premature senility^3^. Oxidative stress-induced mitochondrial DNA mutations can accelerate hepatocyte ballooning degeneration in animal aging models^4^. These results indicate that cell premature aging likely aggravates liver injury. However, the molecular mechanism of cell aging, especially stress-induced premature aging in liver fibrosis, is still poorly understood. Hence, it is important to investigate the underlying mechanism and the intervening target of intrahepatic cell premature senility in liver diseases.

Interestingly, the diverse cell senescence mechanisms underlie biological multifunction. The senescence of intrahepatic cells, including hepatocytes, HSCs, and HSECs, is observed in liver fibrosis^2^. Senescent HSCs, mediated by p53, restrict the progression of liver fibrosis and hepatocellular carcinoma via NK cell-mediated killing^19,20^. Though halting HSC proliferative and collagen-production activated HSC senescence induces more inflammatory cytokines to accelerate liver damage^21^. In our previous studies, we also found that hepatocyte premature senescence emerged in human fibrotic livers and CCl_4_-induced liver injury rat models. During the progression of CCl_4_-induced hepatic steatosis and fibrogenesis in vivo, CCl_4_ induced gradually activation of SA-β-gal, a biomarker of cell senescence, along with a time-dependent elevation of oxidation in hepatocytes. Furthermore, in vitro, H_2_O_2_, confirmed as a golden standard for initiating oxidative stress, aggravated hepatocyte premature senescence. These data indicate that dysfunctional and premature senescent hepatocytes, induced by oxidative stress, likely initiate liver damage and fibrogenesis.

Next, we further explore the mechanisms about stress-induced hepatocyte premature aging. Prelamin A and subsequently mature Lamin A/C protein constitutes the nuclear lamina and nuclear matrix, and involves in a variety of nuclear activities to keep cellular stability and activity^22–24^. Nevertheless, prelamin A and Lamin A/C mutations trigger many distinct of multiple tissues disorder and a series of diseases. Progerin is a kind of mutant prelamin A protein and a special marker of cell premature senescence; moreover, the abnormal accumulation and distribution of progerin is a hotspot for association with cell dysfunction and a variety of diseases^5–7^. Indeed, we also demonstrated that the protein levels of progerin and Lamin A/C, with hepatocyte premature senescence, increased in liver tissues of both cirrhosis patients and CCl_4_-induced rat models. Both in vivo and in vitro, CCl_4_- or H_2_O_2_-induced oxidative stress caused an onset of hepatocyte premature aging; while these effects were rescued by knockdown of p53 with p53 siRNA or knockdown of progerin with progerin siRNA, except knockdown of p21. These data confirm that excess oxidation triggers hepatocyte premature senescence through the p53/progerin pathway, which is the p21-independent pathway.

Besides, IGF-1, a hepatoprotective and growth-promoting hormone which is mainly synthesized and secreted in liver, involves in liver metabolism, tissue repair, and pathogenesis of liver diseases^25–28^. In clinicopathological researches, serum IGF-1 level and tissue expression of IGF-1 decrease in liver diseases, including hepatic steatosis, fibrosis, and cirrhosis; whereas systemic IGF-1 administration can ameliorate liver function and fibrosis^29^. Recombinant IGF-1 (rIGF-1) treatment promoted hepatoprotective activities in cirrhotic patients^11–14^. Gene transfer of IGF-1 increased expression of antifibrogenic molecules and reduced expression of profibrogenic factors to relieve the cirrhotic liver animal models^15^. According to these findings, IGF-1 therapy may be of value for improving liver diseases.

However, the indepth mechanism about ameliorating liver fibrosis with IGF-1 is unknown yet. It is reported that IGF-1 promotes hepatocyte proliferation, differentiation, cell cycle progression, and prolonging survival^30–32^, suggested that IGF-1 might repress cell premature aging and repair intrahepatic cells to ameliorate liver diseases. Nonetheless, some scholars represented different administrations of IGF-1 could tend to promote cell proliferation or premature senescence mediated by p53^10^. To understand the role of exogenous IGF-1 and gene transfer of IGF-1 in stress-induced hepatocyte premature aging, in vivo, IGF-1 gene transferred to CCl_4_-induced rat models with lentivirus vectors to overexpress intrahepatic IGF-1; in vitro, we administered primary hepatocytes with short-term and prolonged exogenous IGF-1, or IGF-1 overexpression with IGF-1 adenovirus vector, and then treated with H_2_O_2_ (200 nM) for 24 h. Our results displayed that prolonged exogenous IGF-1 or IGF-1 overexpression downregulated the p53/progerin pathway to prevent hepatocyte premature senescence. Somewhat surprisingly, in cytoplasm, IGF-1 gene therapy strengthened cytoplasm AKT1–p53 interaction to inhibit H_2_O_2_-initiated p53 nuclear translocation; meanwhile, IGF-1 overexpression, with adenovirus vector, broke nuclear p53–progerin interaction to decrease H_2_O_2_-induced hepatocyte premature senescence.

Altogether, it seems that overexpressing IGF-1 plays a dual role in prevention of oxidative SIPS in hepatocytes: on the one hand, in cytoplasm, IGF-1 activates the PI3K/AKT1 pathway to inhibit p53 nuclear translocation, mediated by the AKT1–p53 interaction; on the other hand, in nuclei, IGF-1 breaks the interaction between p53 with progerin to decrease hepatocyte premature aging.

There are some limitations to the present study. We still have not clearly revealed the mechanism about increased p53 redistribution from cytoplasm to nuclei of stress-induced hepatocytes. Furthermore, the role of IGF-1 in attenuating hepatocyte premature senescence mediated by progerin needs further exploration.

In conclusion, prolonged IGF-1 diminishes hepatocyte premature senescence to relief liver fibrogenesis via disrupting nuclear p53–progerin interaction.

## Experimental methods

### Collection of patient samples

Fibrotic liver biopsy specimens (fibrosis stage: F3–4) were obtained from 24 patients with liver fibrosis. Normal liver specimens were obtained from 16 patients who underwent a partial liver resection for hepatic hemangioma. All patients signed the informed written consent, and the Ethics Committee at the local hospital approved the use of samples.

### Animal experimental design

Sprague–Dawley (SD) rats were provided by the Laboratory Animal Center (Henan University of Chinese Medicine, China) and were approved by the Committee on the Ethics of Animal Experiments of Southern Medical University. Animals were housed under a 12:12 h light/dark cycle at 22–24 °C.

#### Establishment of CCl_4_-induced liver fibrosis rat model

Male SD rats (180–220 g) were subjected to intraperitoneal injection of 40% carbon tetrachloride (CCl_4_)-olive oil solution at 2 ml/kg body weight, twice a week for 28 days. At Days 0, 3, 6, 14, and 28, CCl_4_-induced rats were randomly sacrificed (*n* = 6 per group).

#### The treatment of IGF-1 lentivirus vectors

Besides, to investigate the role of IGF-1 in liver fibrosis, the GFP-IGF-1-lentivirus vector and the GFP-blank vector were produced by Hanbio AdenoVector Institute (Shanghai, China) and the dose of 10^11^ viral particles was injected through caudal vein to rats 1 week before the intraperitoneal injection of CCl_4_-olive oil solution. We employed the CCl_4_-induced liver fibrosis rat models (*n* = 6 for 6 days and *n* = 6 for 28 days). The vehicle group (*n* = 6 for 6 days and *n* = 6 for 28 days) was subjected to intraperitoneal injection of the same volume of olive oil, twice a week for 28 days. The LV-CTR + CCl_4_ group and the LV-IGF-1 + CCl_4_ group (*n* = 6 per group for 6 days and *n* = 6 per group for 28 days) was subjected to intraperitoneal injection of CCl_4_-olive oil solution twice a week after administering vectors. Liver samples were processed for histology and proteins for further analysis.

### Measurement of plasm IGF-1

The plasm IGF-1 of patients and rats were detected by the Human IGF-1 ELISA Kit and the Rat IGF-1 ELISA Kit (Elabscience, E-EL-H0086c and E-EL-R0010c), according to the manufacturer instructions. The results were read and calculated by ELISA.

### Hydrogen peroxide assay

The H_2_O_2_ content in cells or liver tissue was measured by a Hydrogen Peroxide Assay Kit (Beyotime, S0038), and the OD value was detected by absorption spectroscopy (562 nm).

### Measurement of the activity of senescence-associated β-galactosidase (SA-β-gal)

The activity of SA-β-gal in rats and patients liver tissues sections and cultured primary rat hepatocytes was determined using 5-bromo-4-chloro-3-indolyl P3-d-galactoside (X-gal), according to the manufacturer instruction (Senescence-associated β-Galactosidase Staining Kit, Beyotime, C0602). SA-β-gal-positive cells (blue color) were counted under microscope (BX51, Olympus, Japan).

### Histological analysis and immunohistochemistry

Paraffin sections (4 μm) of rat and human liver tissues were prepared with hematoxylin and eosin (H&E) staining and Masson staining. Immunohistochemical detection of p53 and progerin, were performed on paraffin sections (3 μm), and subsequent sections were exposed to horseradish peroxidase (HRP)-antibody colored with DAB, and visualized by microscopy (BX51, Olympus, Japan). The degree of liver fibrosis and the number of p53- or progerin-positive cells were quantified with Image J software.

### Cell isolation, identification, culture, and treatment

Primary rat hepatocytes were isolated from male SD rats, based on the modified method^15^. The isolated hepatocytes were identified by the expression of albumin protein, which was detected by flow cytometry. Primary rat hepatocytes were cultured in plates with medium comprising 80% 1640 (Gibco, 11875101) and 20% fetal bovine serum (FBS, TransSerum, 10102). Primary hepatocytes on Day 2 were stimulated by H_2_O_2_ (30% hydrogen peroxide, 7722-84-1) with a concentration gradient of 0, 10, 50, 100, 200 nM for 24 h, or a time gradient of 0, 1, 6, 12, 24 h, or pretreated with 100 ng/ml IGF-1 (Insulin-like growth factor-1, PeproTech, 081701).

### IGF-1 adenovirus transfection

The recombinant adenovirus was produced by Hanbio AdenoVector Institute (Shanghai, China). To construct Flag protein-tagged IGF-1, fulllength IGF-1 cDNA was amplified from a human cDNA library and fused at its C-terminus with sequences encoding monomeric protein. Briefly, the amplified IGF-1 fragment was inserted into the adenoviral vector, which contains the mouse cytomegalovirus (CMV) promoter, using the AdMax system. The resultant IGF-1-Flag protein gene fusion was validated by nucleotide sequencing. Transfection efficiency, which was assessed by detecting Flag protein using immunoblotting (IB), was 70–80%, respective of the amount of plasmid used in the transfection. Primary hepatocytes were transfected with this adenovirus vector to overexpress IGF-1, according to the manufacturer’s instructions.

### Small interfering RNA (siRNA) transfection assay

Primary hepatocytes were transfected with siRNA to knockdown p53, p21, and progerin, according to the manufacturer instructions. The transfection efficiency was 70%. The following p53 siRNA sequences were used: sense (5-GGCTCCGACTATACCACTA-3). The following p21 siRNA sequences were used: sense (5-GTTGACGATGCCTTCTATA-3). The following progerin siRNA sequences were used: sense (5-GCTCAGTGACTGTGGTTGA-3).

### Extraction of nuclear and cytoplasmic protein of primary hepatocytes

Nuclear and Cytoplasmic Protein Extraction Kit (Beyotime, P0028) was used to extract nuclear and cytoplasmic protein of primary hepatocytes (10^7^ cells/group).

### Co-immunoprecipitation

Primary hepatocytes were transfected with the above adenovirus vector to overexpress IGF-1 or stimulated with H_2_O_2_ (200 nM), following extracted nuclear and cytoplasmic protein. Immunoprecipitation (IP) and western blotting (WB) were performed as previously described^33^. The antibody for IP was anti-p53 and non-specific IgG; the antibodies for WB included anti-p53, anti-AKT1, and anti-progerin.

### Western blotting

Primary hepatocytes were isolated from normal rats and the model rats. Hepatocytes were lysed in lysis buffer containing protease cocktails inhibitor (Beyotime, P1005) and PMSF (Phenylmethanesulfonyl fluoride, Beyotime, ST506), as well as centrifuged at 12000 r/min, 4 °C, for 15 min. The protein expression was detected by western blot. The primary antibodies included anti-progerin (1:50, Santa Cruz, sc-81611), anti-Lamin A/C (1:2000, CST, 4777), anti-p53 (1:1000, Abcam, ab131442), anti-p21 (1:1000, Abcam, ab109199), anti-PI3K (1:1000, CST, 4249), anti-p-AKT1 (S473) (1:1000, Proteintech, 66444-1-Ig), anti-AKT1 (1:1000, CST, 2938), and anti-GAPDH (1:1000, Proteintech, 60004-1). The secondary antibodies were HRP-conjugated Affinipure Goat Anti-Mouse IgG(H + L) (1:10,000, Proteintech, SA00001-1) and HRP-conjugated Affinipure Goat Anti-Rabbit IgG(H + L) (1:10,000, Proteintech, SA00001-2). The protein bands were visualized using the Pierce^™^ ECL Western Blotting Substrate (Thermo, 32106).

### Cell immunofluorescence staining

To reveal p53, AKT1, and progerin proteins in primary hepatocytes, double immunostaining was conducted. Paraformaldehyde-fixed primary hepatocytes were incubated with the primary antibody, followed by the secondary antibody, and subsequently mounted with DAPI. The primary antibodies included anti-p53 (1:200, mouse, Abcam, ab131442), anti-p53 (1:200, rabbit, Proteintech, 10442-1-AP), anti-AKT1 (1:200, rabbit, CST, 2938), and anti-progerin (1:25, mouse, Santa Cruz, sc-81611). The number of positive cells was observed by fluorescence microscopy (1 × 71, Olympus, Japan) and quantified by Image J software.

### Statistics

The data were reported as the mean ± SD and were analyzed by SPSS17.0 software. In statistical analysis of two groups, a two-tailed Student’s *t* test was utilized; whereas, in statistical analysis of more than two groups, one-way ANOVA was performed.

## Supplementary information

Supplement

Supplementary Figure 1

Supplementary Figure 2

Supplementary Figure 3

Supplementary Figure 4

Supplementary Figure 5
