# Supplementary material for: Insulin-like growth factor-1 attenuates oxidative stress-induced hepatocyte premature senescence in liver fibrogenesis via regulating nuclear p53–progerin interaction
Source: Cell Death Dis. 2019 Jun 6;10(6):451. doi: 10.1038/s41419-019-1670-6 (PMC6554350; doi:10.1038/s41419-019-1670-6)
Supplement: Supplementary file 1 — Supplement [file 41419_2019_1670_MOESM1_ESM.docx]

**Supplement:**

**
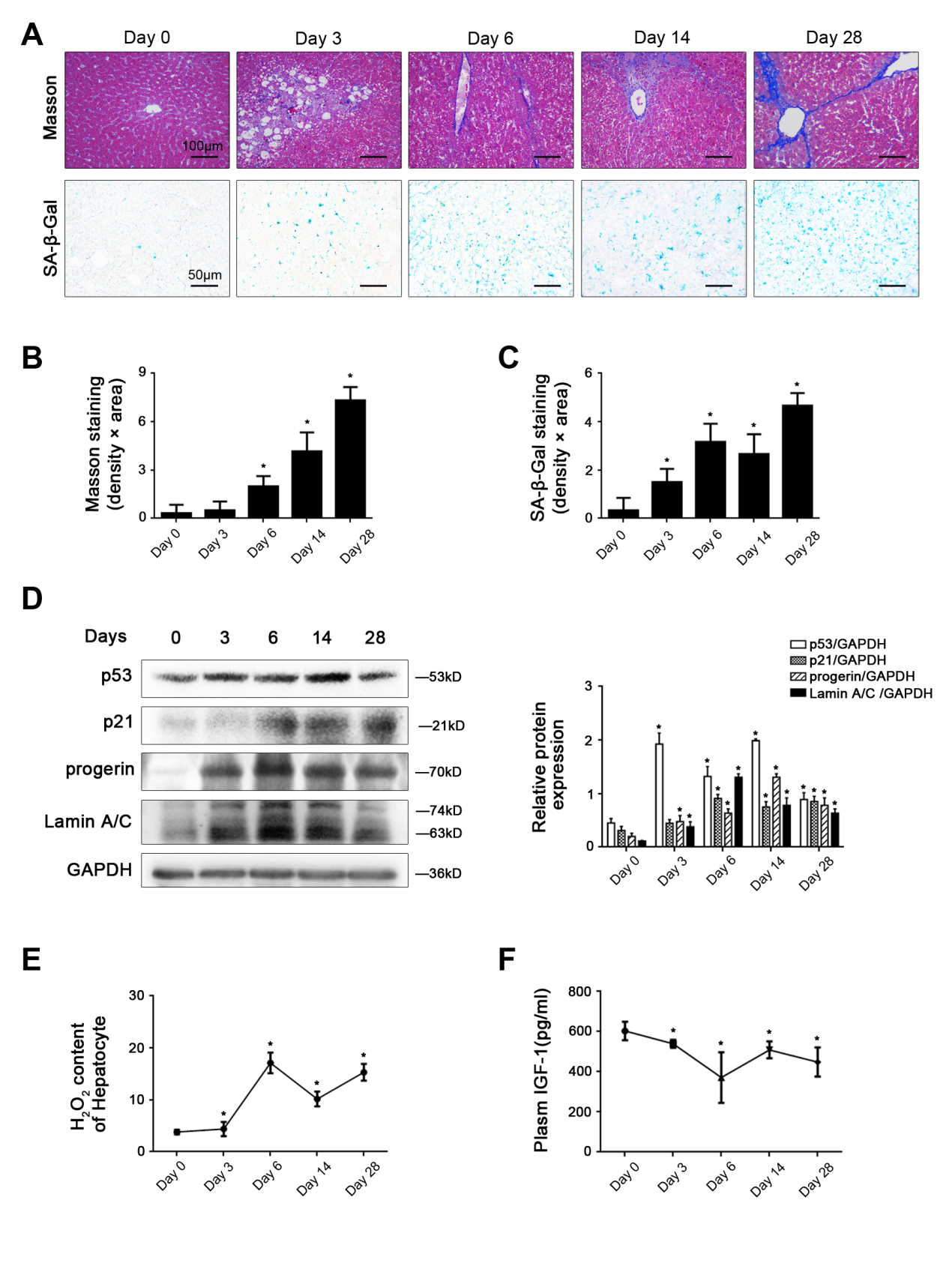
**

**Supplementary Figure 1 Hepatocyte premature senescence is initiated by oxidative stress, along with reduction of IGF-1 during CCl_4_-induced liver fibrogenesis.** (A) Masson staining and SA-β-gal staining in liver biopsy specimens of CCl_4_-induced rat models (Day 0, Day 3, Day 6, Day 14, and Day 28) (Scale bar: 100 μm, 50 μm). (B) The area density of Masson staining. ^*^P<0.05 versus Day 0. (C) The area density of SA-β-gal staining. ^*^P<0.05 versus Day 0. (D) Representative immunoblots of p53, p21, progerin, and Lamin A/C of primary hepatocytes isolated from the CCl_4_-induced rat models. The relative protein expression is quantified in the graph, right. ^*^P<0.05 versus Day 0. (E) The H_2_O_2_ content of primary hepatocytes isolated from the CCl_4_-induced rat models. ^*^P<0.05 versus Day 0. (F) The plasm IGF-1 content in CCl_4_-induced rat models. ^*^P<0.05 versus Day 0. n=6 per group.

**
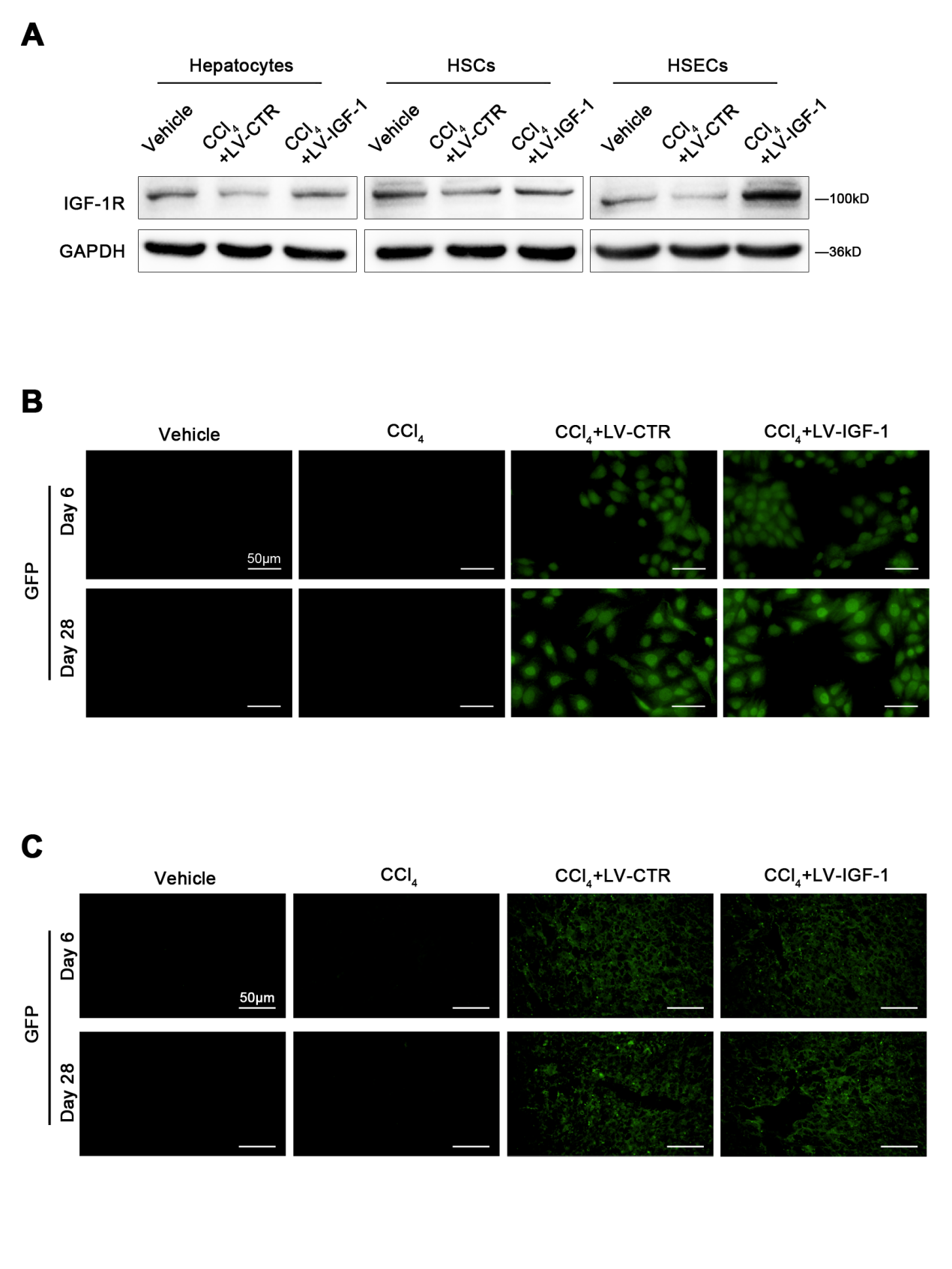
**

**Supplementary Figure 2 IGF-1 gene transfer to CCl_4_-induced rat models elevated the IGF-1R expression of intrahepatic cells.** Freshly hepatocytes, hepatic stellate cells (HSCs), and hepatic sinus endothelial cells (HSECs) isolated from the CCl_4_-induced rat models on Day 28. (A) Representative immunoblots of IGF-1R in primary rat hepatocytes, HSCs, and HSECs in the CCl_4_-induced rat models on Day 28. (B) Lentivirus vectors were showed by GFP (green). The GFP expression (green) in primary hepatocytes of rat models on Day 6 and Day 28. (C) The GFP expression (green) in liver tissue of rat models on Day 6 and Day 28.

**
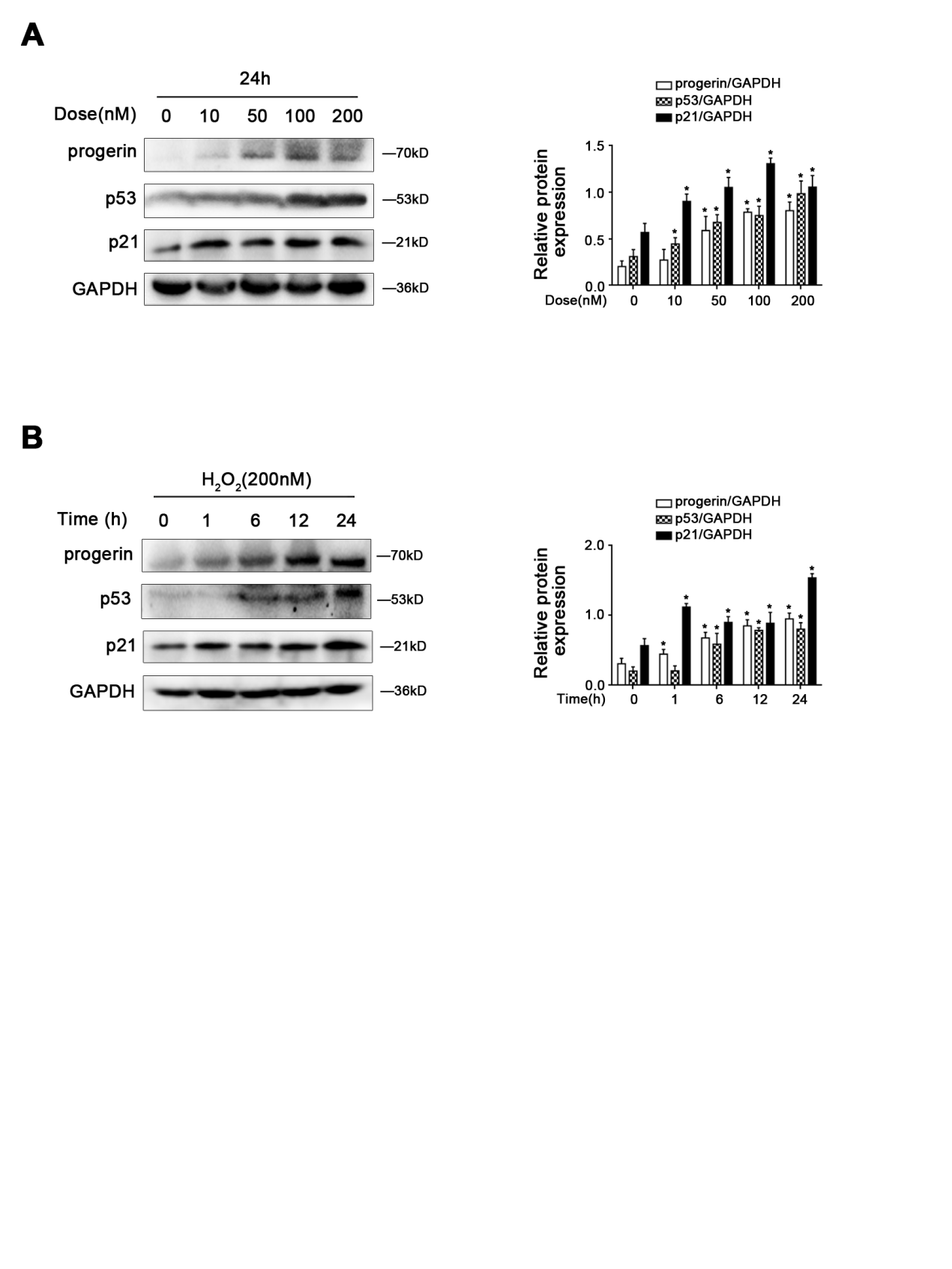
**

**Supplementary Figure 3 H_2_O_2_ up-regulated the protein level of progerin, p53, and p21.** Freshly primary hepatocytes, isolated from normal rats, were cultured and stimulated with H_2_O_2_ at the different doses (0, 10, 50, 100, 200 nM) for 24 hours or at the dose (200 nM) from 0 to 24 hours. (A) Representative immunoblots of progerin, p53, and p21 of primary rat hepatocytes. The relative protein expression is quantified in the graph, right. ^*^P<0.05 versus the 0 nM group. (B) Representative immunoblots of progerin, p53, and p21 of primary rat hepatocytes. The relative protein expression is quantified in the graph, right. ^*^P<0.05 versus the 0 h group.


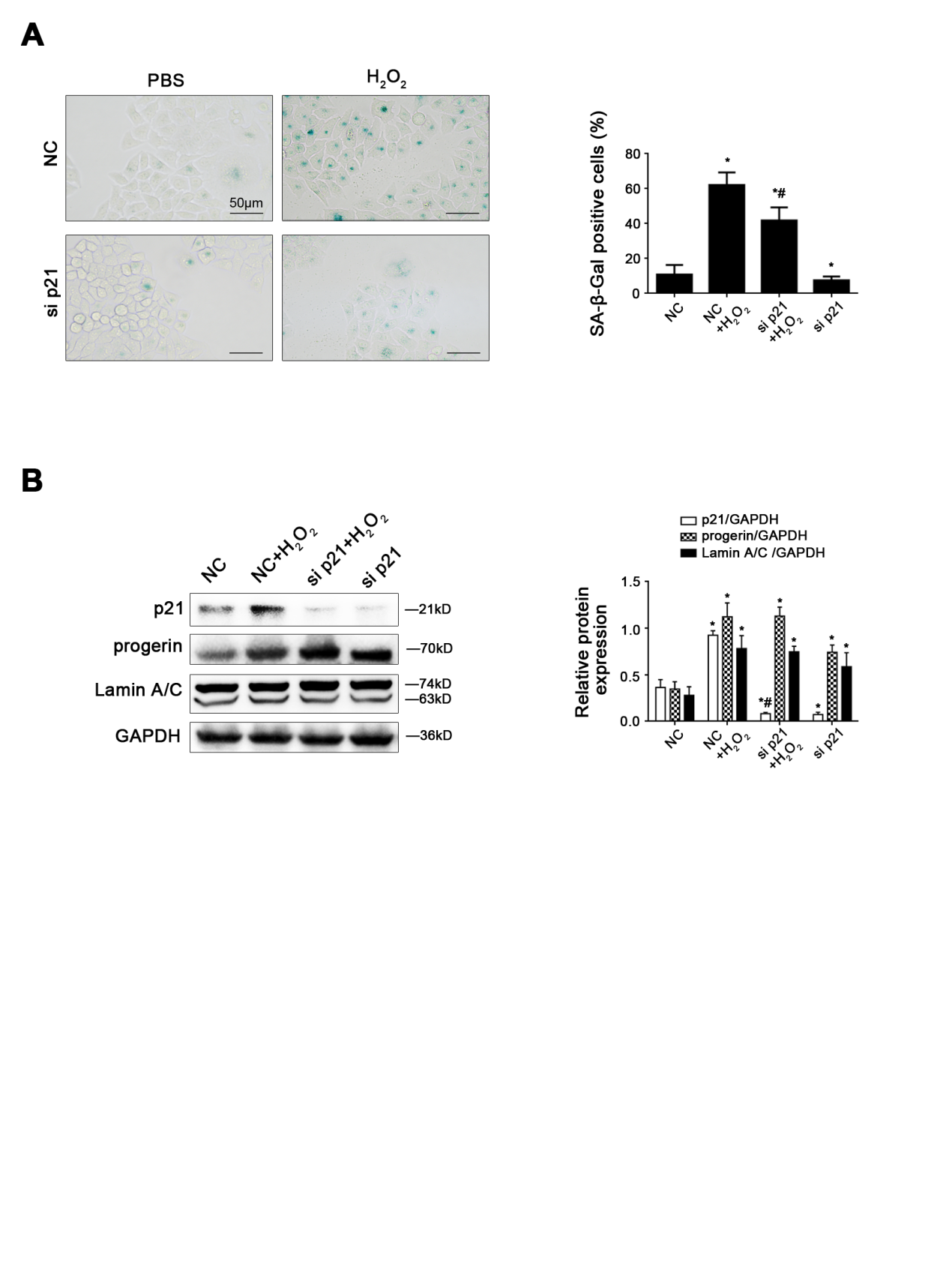


**Supplementary Figure 4 H_2_O_2_ induced hepatocyte senescence via the p21-dependent pathway.** Freshly primary hepatocytes, isolated from normal rats and cultured *in vitro*, were transfected with p21 siRNA or nontarget siRNA (called NC), and then administered with H_2_O_2_ (200 nM) for 24 hours. (A) SA-β-gal activity in rat hepatocytes of the four groups (NC, NC+H_2_O_2_, H_2_O_2_+si p21, si p21), was revealed by SA-β-gal staining. Scale bar: 50 μm. SA-β-gal positive cells are quantified in the graph, right. ^*^P<0.05 versus the NC group; ^#^P<0.05 versus the NC+H_2_O_2_ group. (B) Representative immunoblots of p21, progerin, and Lamin A/C of primary rat hepatocytes. The relative protein expression is quantified in the graph, right. ^*^P<0.05 versus the NC group; ^#^P<0.05 versus the NC+H_2_O_2_ group.


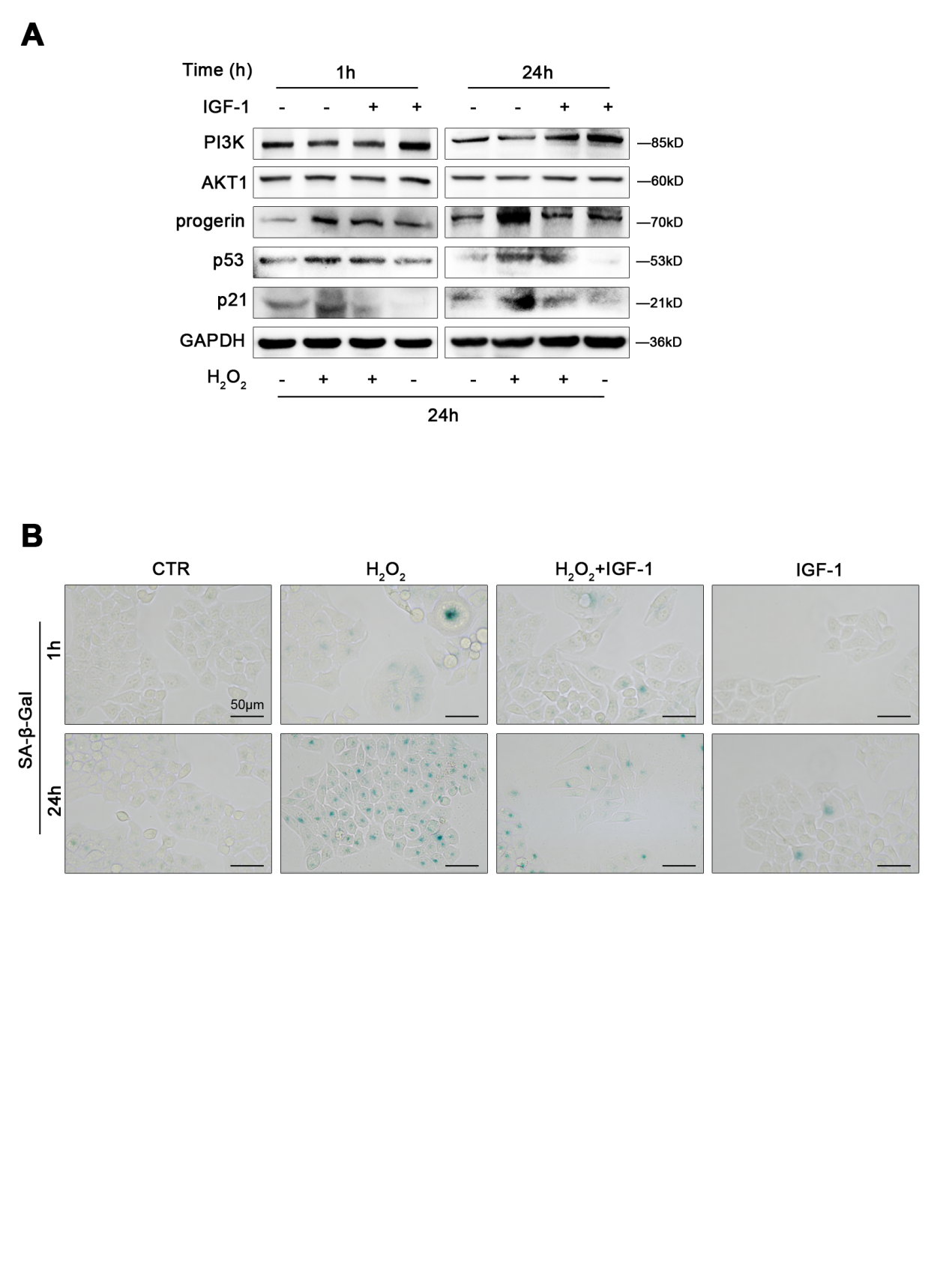


**Supplementary Figure 5 Short-term and prolonged exogenous IGF-1 regulated hepatocyte senescence through activating the PI3K/AKT1 pathway.** Primary hepatocytes, isolated from normal rats, treated with H_2_O_2_ (200 nM), were administered with IGF-1 (100 ng/ml) for 1 hour and 24 hours simultaneously. (A) Representative immunoblots of PI3K, AKT1, p53, and p21 of primary rat hepatocytes. (B) SA-β-gal activity in rat hepatocytes was revealed by SA-β-gal staining. Scale bar: 50 μm.
