# Supplementary figures and images for: Insulin-like growth factor-1 attenuates oxidative stress-induced hepatocyte premature senescence in liver fibrogenesis via regulating nuclear p53–progerin interaction
Source: Cell Death Dis. 2019 Jun 6;10(6):451. doi: 10.1038/s41419-019-1670-6 (PMC6554350; doi:10.1038/s41419-019-1670-6)

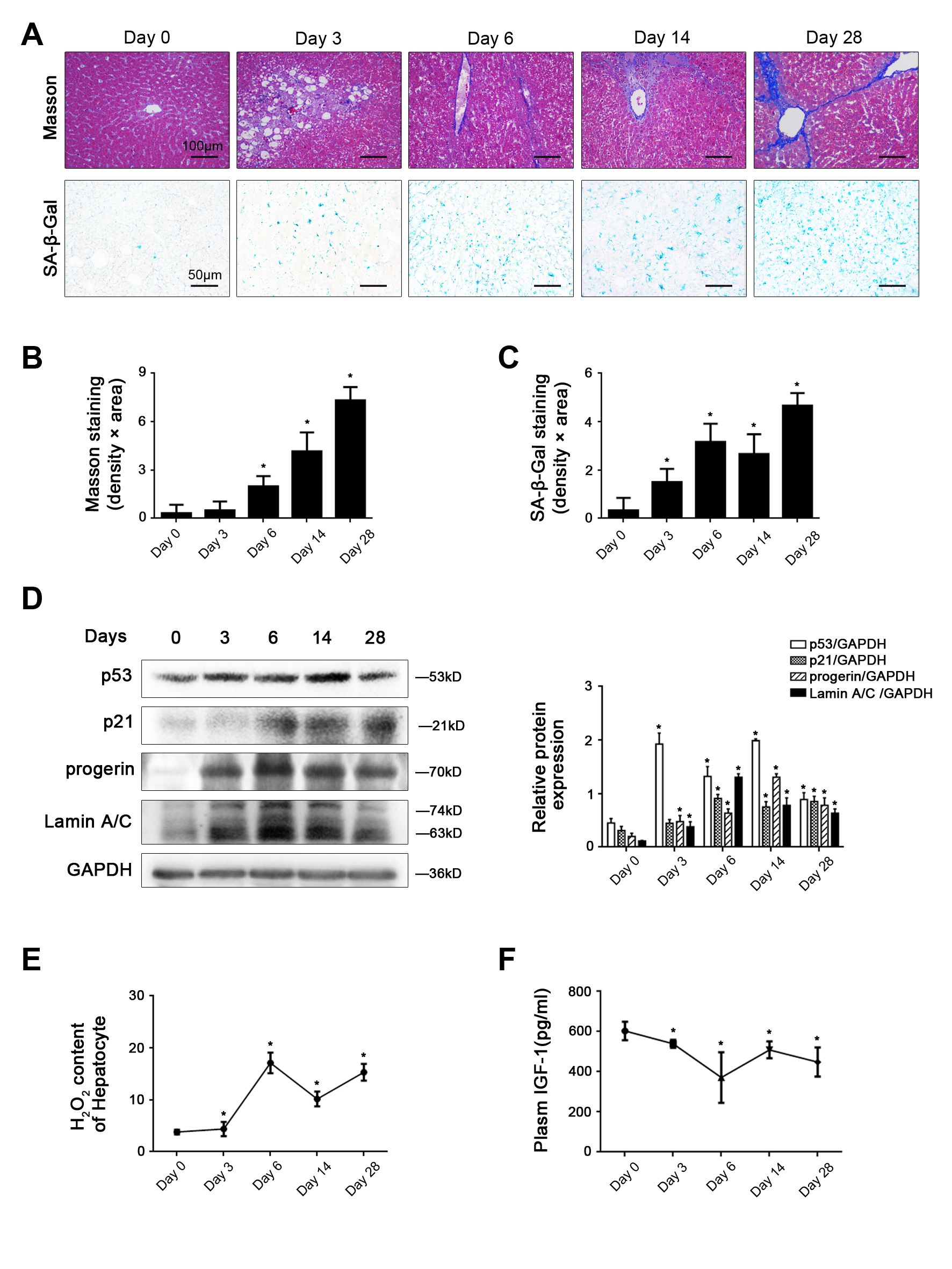

Supplement: Supplementary file 2 — Supplementary Figure 1 [file 41419_2019_1670_MOESM2_ESM.tif]

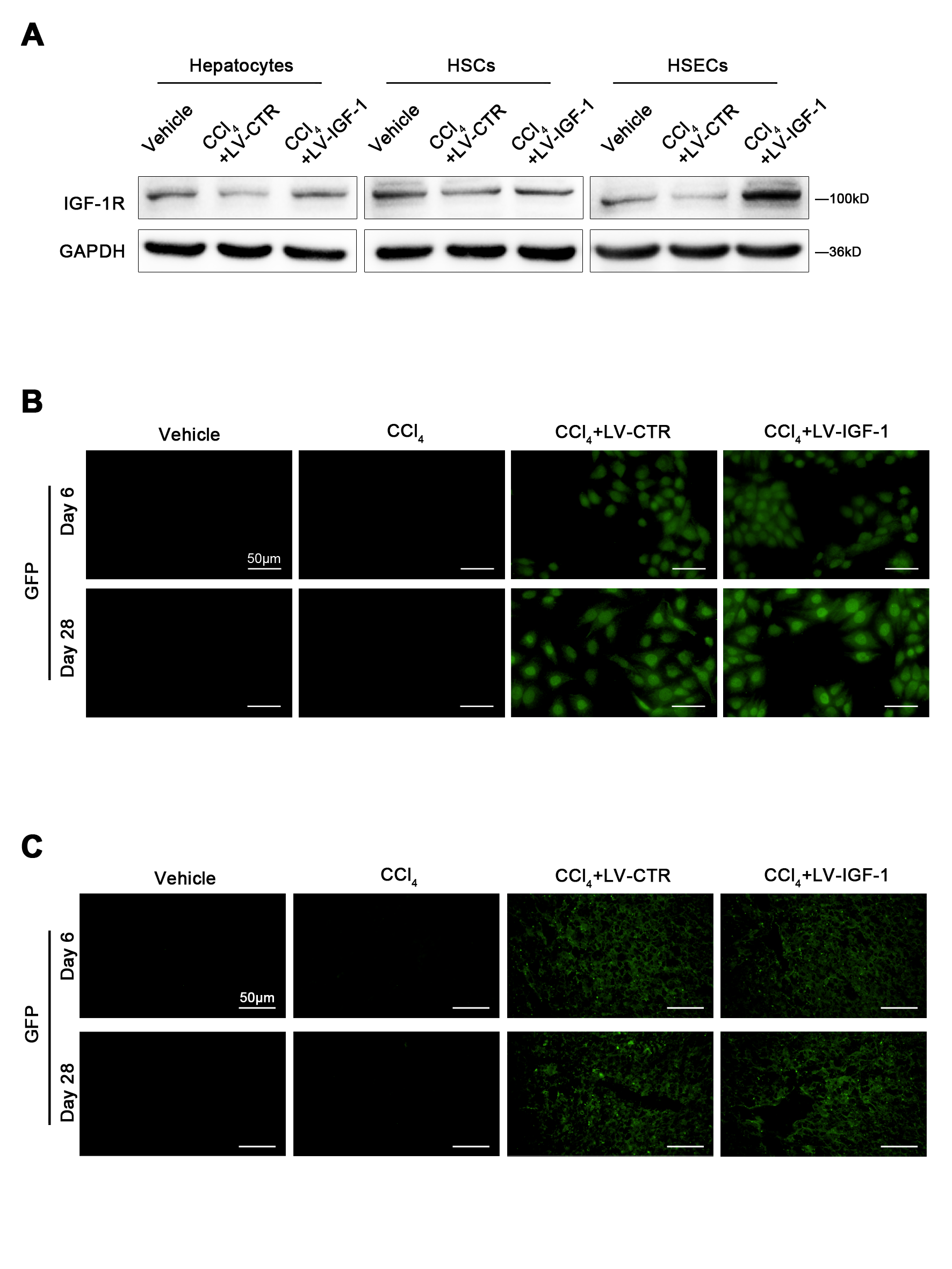

Supplement: Supplementary file 3 — Supplementary Figure 2 [file 41419_2019_1670_MOESM3_ESM.tif]

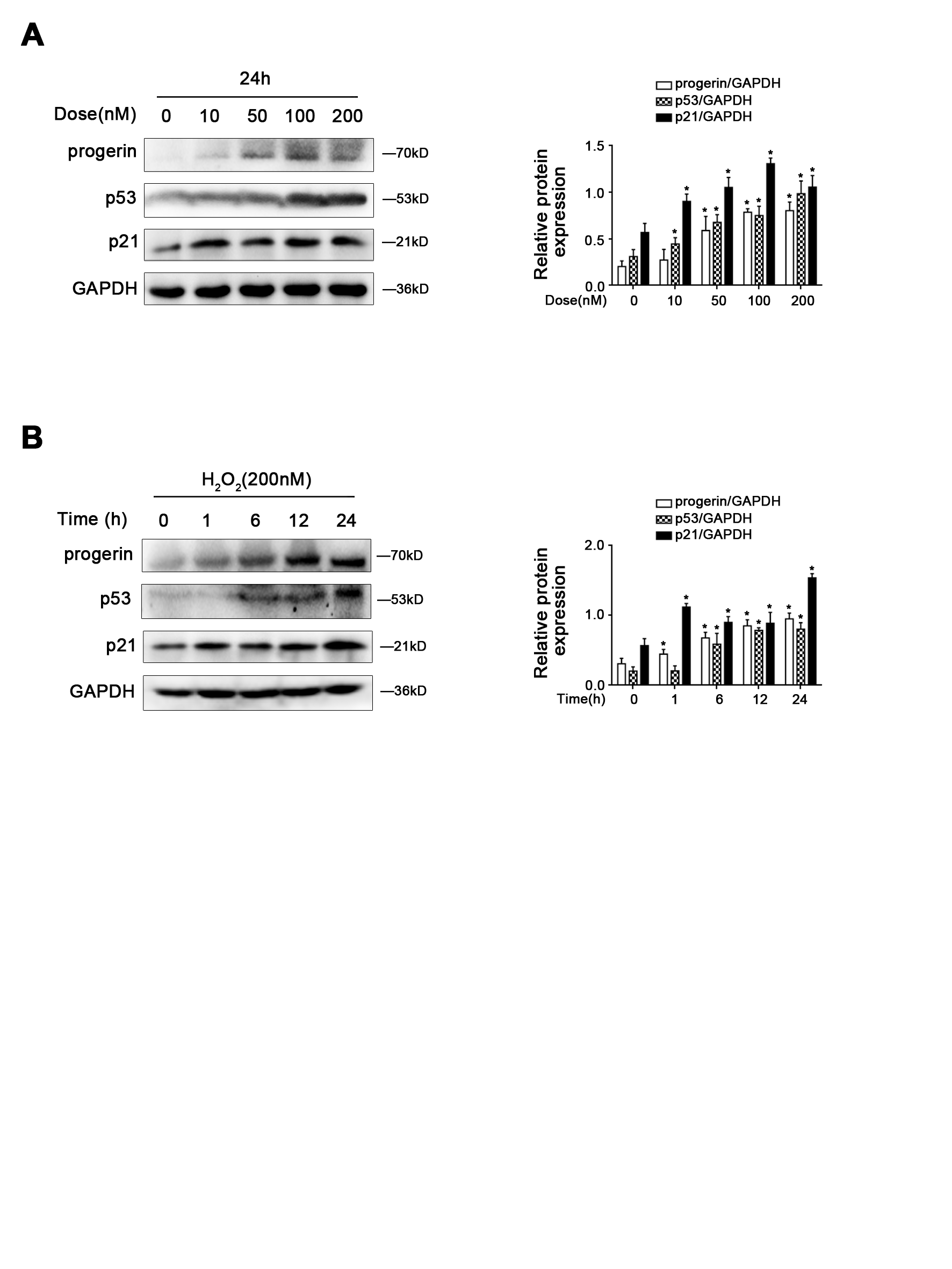

Supplement: Supplementary file 4 — Supplementary Figure 3 [file 41419_2019_1670_MOESM4_ESM.tif]

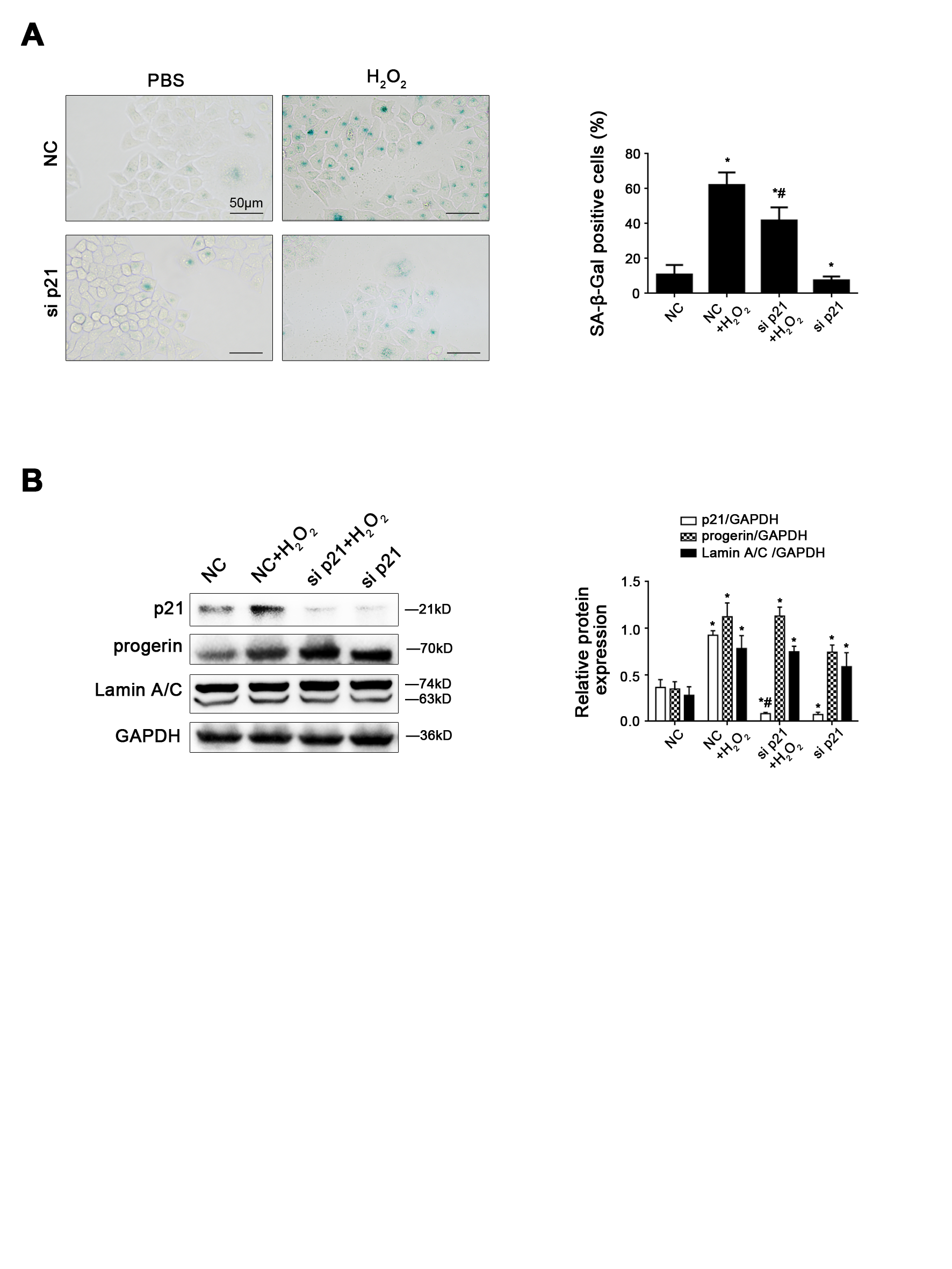

Supplement: Supplementary file 5 — Supplementary Figure 4 [file 41419_2019_1670_MOESM5_ESM.tif]

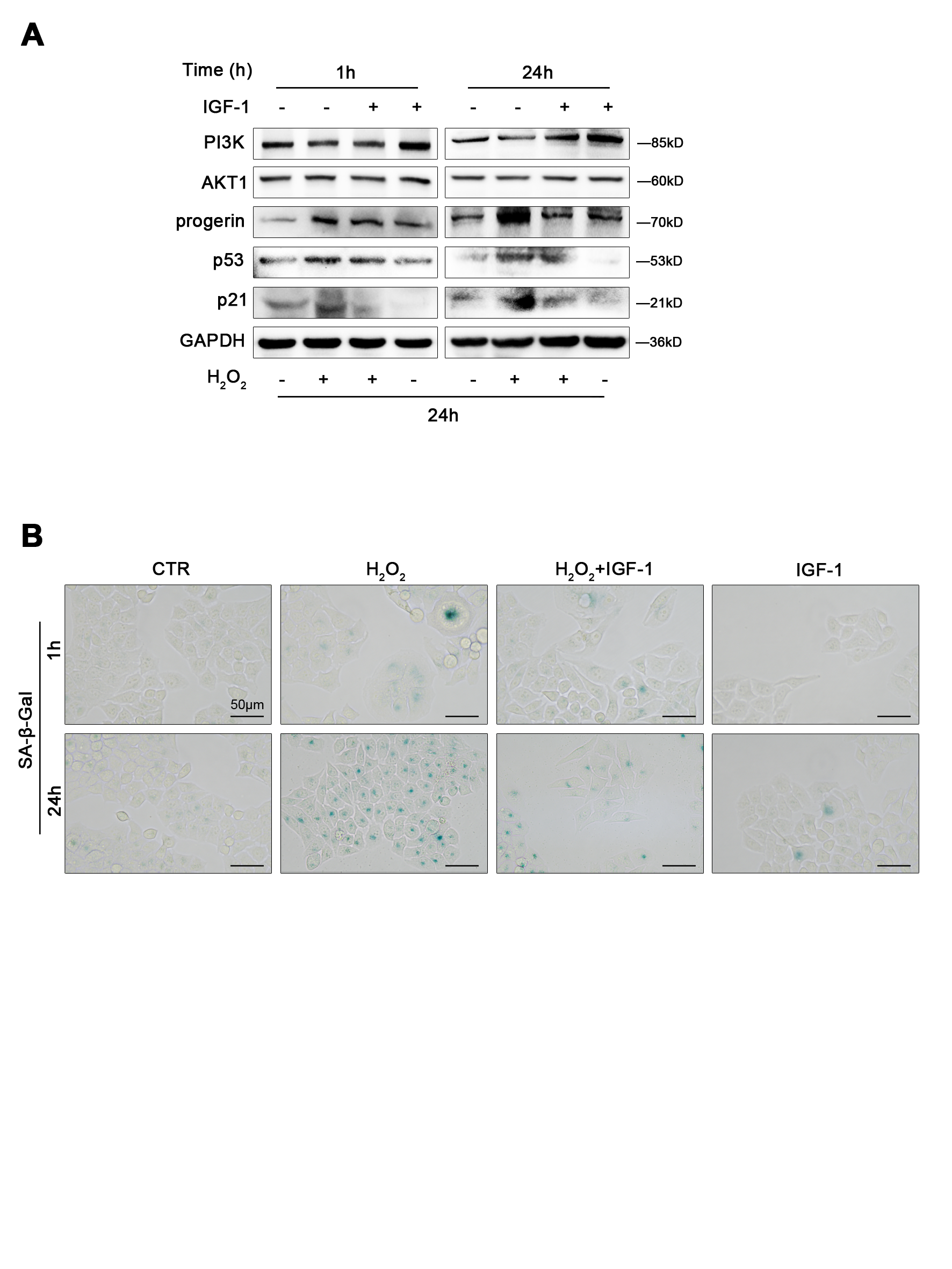

Supplement: Supplementary file 6 — Supplementary Figure 5 [file 41419_2019_1670_MOESM6_ESM.tif]
